# Differences in endocrine and reproductive responses to substance exposure across generations: highlighting the importance of complementary findings

**DOI:** 10.1007/s00204-024-03813-3

**Published:** 2024-07-18

**Authors:** Ingo Bichlmaier

**Affiliations:** European Chemicals Agency, Hazard Assessment Directorate, Telakkakatu 6, 00150 Helsinki, Finland

**Keywords:** Endocrine toxicity, Reproductive toxicity, Association, Occurrence of effects, Similarity, Generational response, Jaccard coefficient, Venn diagram

## Abstract

This article analyzes the results from 112 Extended One-Generation Reproductive Toxicity studies. The objective was to determine if test animals show consistent endocrine and reproductive effects within the same and across different generations and life stages. The analysis, grounded in a comprehensive Binary Matrix, included 530 observed effects and 193 unique, statistically significant associations. Associations’ strength was quantified using Jaccard (***J***) coefficients to measure effect co-occurrence in the same study. Associated effects co-occur infrequently across the whole dataset (median ***J*** = 0.231). However, specific patterns emerged: associations of same effects across generations exhibited a higher strength (median ***J*** = 0.400) compared to associations of different effects (median ***J*** = 0.222). Notably, associations with effects observed in both the parental animals of the adult first filial generation (P1) and developing second filial generations (dF2) demonstrated ***J*** coefficients (with medians ranging from 0.300 to 0.430) that were approximately twofold higher than those of other associations. Consistently, equivalent life stage associations across generations revealed statistically significant higher association strengths for the P1 and dF2 generations (median*s* of 0.375 and 0.333, respectively) compared to other generations (medians of 0.200 and 0.174), possibly due to longer exposure duration and altered cross-talk between pregnant P1 dam and its conceptus. Overall, it is concluded that co-occurrence of associated effects in the same study is rather infrequent and that associations with effects in P1 and dF2 are stronger than all other associations. In general, the findings underscore the importance of independently analyzing each effect per generation due to the generally low co-occurrence rates of associated effects, challenging traditional expectations of generational continuity in toxic effects.

**Supplementary Information:**

The online version contains supplementary material available at 10.1007/s00204-024-03813-3.

## Highlights

- Identifying endocrine and reproductive toxicants is crucial for safeguarding the ability for humans to reproduce, as well as for protecting human embryos, fetuses, and children.
- This objective requires that the intrinsic toxic properties related to endocrine and reproductive health are thoroughly investigated.
- 112 recently conducted Extended One-Generation Reproductive Toxicity studies were evaluated and analyzed for endocrine and reproductive toxicity.
- The results show that it is much more common to observe endocrine/reproductive effects in only one generation, but not in another, within the same study (degrees of co-occurrence as low as approximately 9%).
- Associations with effects in the parental animals of the adult first filial generation and/or developing second filial generation are significantly stronger than all other associations (degrees of co-occurrence as high as approximately 49%).
- The necessity of independently analyzing each endocrine and reproductive effect within each generation is highlighted. Observed endocrine and reproductive effects should not be disregarded simply because they occur in one generation but not in another.

**Supplementary Information:**

The online version contains supplementary material available at 10.1007/s00204-024-03813-3.

## Introduction

The Extended One-Generation Reproductive Toxicity (EOGRT) study, as outlined in OECD Test Guideline (TG) 443, was adopted in 2011 and subsequently revised in 2012 and 2018 (OECD 2018a). This advanced in vivo study shares similarities with the Pre- and Post-Natal Development Study, including aspects of Maternal Function, in accordance with the guidelines established by the International Council for Harmonisation of Technical Requirements for Pharmaceuticals for Human Use (ICH 2020).

The EOGRT study examines sexual function, fertility, and development across several life stages: pre-mating, mating, gestation, parturition, weaning, and post-weaning up to adulthood. These stages are investigated in the initial adult parental generation producing the first filial generation, with an option to extend the study from the first to the second filial generation. The study evaluates structural changes and functional alterations in sexual function and fertility, as well as development. Moreover, it investigates highly endocrine-sensitive parameters to elucidate possible endocrine mechanisms behind observed changes. These parameters include nipple/areola retention, anogenital distance, and indicators of onset of puberty/sexual maturation, meaning balano-preputial separation, vaginal opening, first estrus, and the time between vaginal opening and first estrus, which suggest interference with androgenic or estrogenic pathways (OECD 2018a; Schwartz et al. 2019; Schwartz et al. 2021).

The EOGRT study is regarded as the most thorough method for determining if a chemical meets the World Health Organization/International Programme on Chemical Safety (WHO/IPCS 2002) definition of an endocrine disruptor (OECD 2018b). Notably, the EOGRT study is crucial for assessing the hazardous properties of chemicals under the REACH Regulation (European Parliament and Council 2006) and Biocidal Products Regulation (European Parliament and Council 2012) in the European Union. Since its incorporation into the REACH Regulation in March 2015 (European Commission 2015), the European Chemicals Agency (ECHA) has mandated EOGRT studies for hazard and risk assessments of substances, with approximately 400 studies requested to date. With an estimated average cost of 900,000 EUR per study, this represents a significant investment of around 360 million euros, or approximately 390 million US dollars.

The critical need to protect human health from reproductive and endocrine hazards posed by chemicals is well-documented (WHO/IPCS 2002; La Merrill 2020; Duh-Leong et al. 2023; Hellsten et al. 2023), underscoring the importance of continuous research and regulation in this field. Identifying endocrine and reproductive toxicants is essential for preserving the reproductive capabilities of humans and for the protection of human embryos, fetuses, and children. The comprehensive investigation of toxic properties affecting endocrine and reproductive health is necessitated for this objective. Despite comparative analysis being considered a standard tool in toxicology evaluations, the frequency of intra- and intergenerational occurrence of endocrine and reproductive effects within the same reproductive toxicity study is not well understood.

A report published by the ECHA in 2023 highlights the utility of the EOGRT study in identifying substances that impact sexual function, fertility, development, and endocrine activity. The study contributes significantly to safeguarding the health of parents and their children, both unborn and born, against chemical hazards (ECHA 2023a). A table summarizing the EOGRT study's investigations can be found in the report’s annex (ECHA 2023b).

Approximately 80% of the evaluated studies that reported positive findings were noted to include dismissals of endocrine and reproductive effects as spurious, irrelevant, insignificant, equivocal, not treatment-related, or not adverse. This was justified by the observation of effects only in one generation or life stage but not in another within the same study.

Based on this continuity assumption, the hypotheses posited for this article are as follows:

1. Associated treatment-related endocrine and reproductive effects demonstrate a high level of co-occurrence across different life stages and generations.
2. There are no significant differences in the strength of these associations.

A total of 112 EOGRT studies, finalized between 2016 and 2023 and involving structurally diverse high-tonnage chemicals representing a volume of approximately 20 million tons per year globally, were evaluated and analyzed (Table S1 of the Supplementary Information). The variety of chemical structures and observed effects, standardized under a consistent test protocol and evaluation, is considered to provide a solid foundation for comparative analyses. The studies were subjected to normalization by the consistent evaluation of results to improve utility, such as excluding datasets with redundant information (duplicate reporting) and harmonizing the database to enable identification of the same effects based on different terminologies used. Associations and their logical coherency within and across different life stages and generations were assessed.

## Methods

**Fig. 1:**
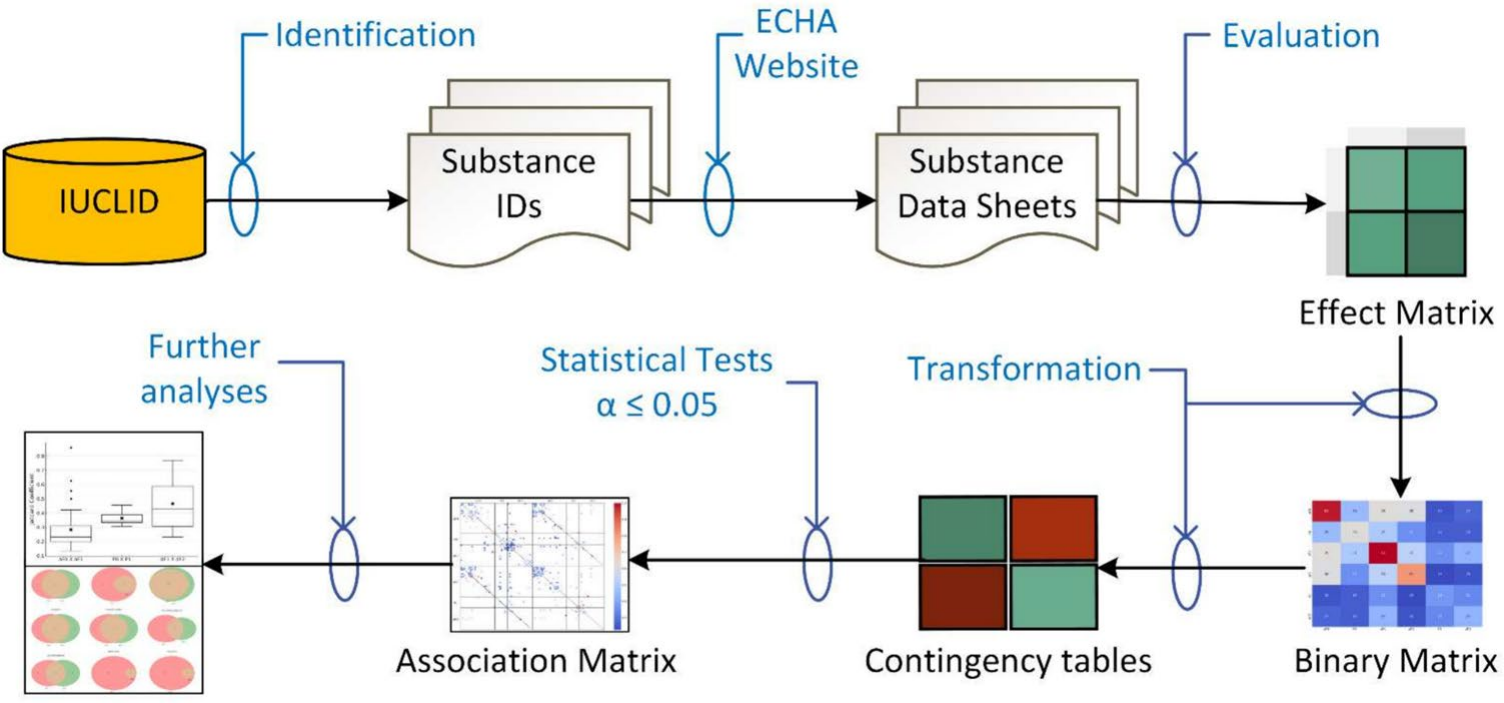
Workflow for identifying public registration dossiers (Substance Data Sheets) from IUCLID (International Uniform Chemical Information Database) that include results from EOGRT studies, evaluating them, recording the findings in the Effect and Binary Matrices, and conducting statistical analyses at the significance level *α* ≤ 0.05, based on 2 × 2 contingency tables. The output files include the Association Matrix, Venn diagrams, and boxplots

The methodology employed is illustrated in Fig. 1. All registration dossiers that included results from EOGRT studies submitted to the ECHA by April 2023 were identified. To avoid conflicts with confidentiality and ensure open access to the dataset, only dossiers publicly available were selected for the evaluation of study results. These dossiers, available on ECHA’s dissemination website, are referred to as Substance Data Sheets.^1^ The investigation’s scope was confined to examining relevant parameters affecting male and female reproduction across various life stages and generations, such as mating, gestation, parturition, lactation, weaning, and post-weaning, in addition to specific endocrine parameters, as detailed in Table S2 of the Supplementary Information.

The results for the selected parameters from the 112 identified EOGRT studies were evaluated (Table S1 of the Supplementary Information). Effects related to the treatment were recorded in a Microsoft Excel spreadsheet, known as the Effects Matrix. An effect was considered related to the treatment if it was statistically significantly different from control values, biologically relevant, demonstrated a dose–response relationship, and/or could not be attributed to secondary, non-specific effects of general toxicity.

The investigations were labeled according to the generation and life stage of the test animals: adult initial generation (aF0), parental animals of the adult initial generation (P0), developing first filial generation (dF1), adult first filial generation (aF1), parental animals of the adult first filial generation (P1), and developing second filial generation (dF2). These labels are also referred to as 'Generation and Life Stage (GALS) groups’ within this article.

This labeling was used to better account for the different investigations carried out on these different animal groups: aF0 and aF1 include investigations on adult animals after sexual maturation without mating, gestation, parturition, and lactation; P0 and P1 cover investigations on mating, gestation, parturition, and pregnancy outcome; dF1 and dF2 encompass investigations on offspring from birth to sexual maturation or weaning, respectively. This GALS grouping supported the analyses performed as it allowed for distinct investigations of associations across different generations and life stages. Therefore, the terminology used in this article differs from that in the EOGRT study protocol which only uses P0, F1, and F2 to explain the study requirements for these different generations (OECD 2018a). Table S2 of the Supplementary Information specifies the investigations conducted on the different GALS groups.

The initial step in the methodology involved converting the Effects Matrix into the Binary Matrix (refer to Fig. S1 in the Supplementary Information), to indicate the presence (1) or absence (0) of specific effects across the 112 EOGRT studies (68 studies provided results only on aF0, P0, dF1 and dF2, 44 studies additionally on P1 and dF2).

The co-occurrence of two effects within a study was compared to the co-occurrence of the same two effects in different studies within the matrix. First, pairs with statistically significant associations according to Fisher’s Exact Test were identified. Second, the Jaccard Similarity Coefficient (***J***) then quantified the degree of similarity between sets that had already been established as significantly associated. While Fisher’s Exact Test determines the presence of a significant association, the ***J*** coefficient quantifies the extent or strength of this association in terms of the co-occurrence of associated effects (refer to the following sections). Additionally, the biological coherence among the identified pairs of associated effects was examined to ensure consistency and relevance.

Effects observed in four or fewer studies were excluded from this analysis due to the insufficient sample size for producing meaningful insights, potentially leading to false positive associations. This cut-off was based on the general rule that insufficient numbers might result in unreliable results for association analyses (Pagano et al. 2022).

Statistically significant associations, determined at the commonly accepted significance level of *α* ≤ 0.05, were identified by applying Fisher’s Exact Test (based on 2 × 2 contingency tables) across all possible pairs of effects—each pair representing a unique combination of two effects—within the entire binary matrix. The Fisher’s Exact Test was chosen because it precisely determines statistical significance. It is non-parametric and does not require the assumption of a normal distribution of the data. The two-sided Fisher’s Exact Test was used to examine if one effect is more likely to occur in the presence of another or vice versa. It allows for detecting any association, regardless of its direction (Pagano et al. 2022). All *p*-values mentioned in this article are derived from the two-sided Fisher’s Exact Test.

The ***J*** coefficient was chosen to investigate the research hypothesis because it measures the rate at which two associated effects co-occur. This makes it a particularly relevant statistical parameter for the research question of this article. It is typically used to explore the similarity between categories. In the context of this project, this means that different generations and life stages are considered more similar when they exhibit similar endocrine and reproductive effects. The ***J*** coefficient is calculated as follows:

$$J= \frac{\left|A\cap B\right|}{\left|A\cup B\right|}.$$

Here, ∣A ∩ B∣ represents the number of studies where both effects co-occur, while ∣A ∪ B∣ is the number of studies where at least one of the effects occurs. A value of 0 indicates no overlap between the studies, meaning no study features both effects. Conversely, a value of 1 signifies that all studies exhibit both effects. Values between 0 and 1 indicate a varying degree of overlap, with higher values reflecting a greater extent of co-occurrence between the two effects.

Venn diagrams are incorporated because they are intuitively understood and effectively visualize the distribution of associated effects within each category. They illustrate both individual occurrences of an effect and the co-occurrence of associated effects. Thus, this visualization proves to be suitable for addressing the research question as it illustrates effect occurrences in the studies. The area of overlap in the Venn diagram indicates the number of effects that appear together in both categories, a measure referred to as the degree of co-occurrence (DCO). In this study, the figures within the Venn diagrams are expressed as percentages relative to the number of total occurrences of each effect, providing a clear representation of the extent to which effects co-occur.

The statistical analyses were conducted using Python libraries, including SciPy and NumPy. The SciPy library facilitated Fisher’s Exact Test, while NumPy was employed for calculating ***J*** coefficients. The results were visualized using Python libraries such as Matplotlib (boxplots), Matplotlib_venn (Venn diagrams), and Seaborn (heatmaps).

## Results

### Binary matrix, associations, and biological coherence

#### Binary matrix

The binary matrix, depicting the presence or absence of endocrine and reproductive effects in EOGRT studies, encompasses 85 × 112 cells (equivalent to 85 investigations × 112 EOGRT studies) and catalogs 530 treatment-related effects. This matrix is illustrated in Fig. S1 of the Supplementary Information.

The heatmap and the boxplot in Fig. S2 of the Supplementary Information depict the total number (prevalence) and distribution of all 530 observed effects at the level of the GALS groups. On average, each effect was observed in 27 studies per group across the entire binary matrix, with a standard deviation of about 13, a minimum occurrence in 14 studies, and a maximum occurrence in 63 studies.

At the level of the individual investigations, the most frequent findings included sperm morphology (34 studies), sperm motility (27 studies), irregular estrus cyclicity (28 studies), pre-implantation loss (27 studies), decreased number of delivered pups (37 studies), post-natal loss (33 studies), delayed balano-preputial separation (28 studies), and delayed vaginal opening (30 studies).

Such a high number of observations (*N*) results in more robust statistical analyses because this large dataset provides a better representation of the underlying population, reducing sampling error and increasing the statistical power, reliability, and validity of the statistical estimates. However, substances with mode of actions underrepresented in the dataset cannot achieve statistical significance due to their low *N*. Therefore, the provided analysis cannot identify all existing associations between effect pairs.

#### Associations of effects in investigations

**Fig. 2:**
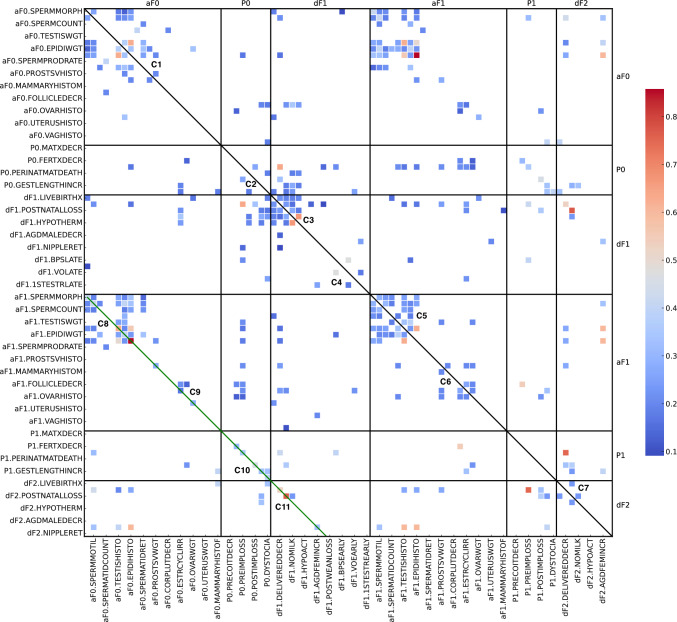
Pairwise associations of the observed effects for all investigations are illustrated in this heatmap. It illustrates pairwise effect-effect associations within and across different generations and life stages: initial adult animals (aF0), the parental animals of the aF0 generation (P0), their developing offspring (dF1) and adult offspring (aF1), the parental animals of the aF1 generation (P1), and their developing offspring (dF2). Association is measured in the form of the Jaccard (***J***) coefficient, which identifies how often associated effects co-occur in the same study compared to all occurrences. Only ***J*** coefficients with *p* ≤ 0.05 are displayed, based on Fisher’s Exact test. Red data points indicate ***J*** coefficients with values closer to 1.0, representing very strong associations. Darker squares signify ***J*** coefficients with lower strengths. The heatmap is divided into segments by black horizontal and vertical lines, indicating the generations and life stages aF0, P0, dF1, aF1, P1, and dF2 (refer to the top x-axis and right y-axis). The black diagonal line divides the heatmap into two symmetrical parts, the upper and lower triangles. Data points in segments divided by the black diagonal represent effect-effect associations within the same generation, meaning aF0 × aF0, P0 × P0, dF1 × dF1, aF1 × aF1, P1 × P1, and dF2 × dF2. Data points in segments not divided by this black diagonal represent associations across different generations and life stages, for example, aF0 × P0, aF0 × dF1, aF0 × aF1, aF0 × P1, and aF0 × dF2. Data points on the green diagonal represent associations in identical investigations (same effects) between different generations of the same life stages, meaning aF0 × aF1, P0 × P1, and dF1 × dF2. C1 to C11 identify clusters of associations. The total number of unique data points is 193, with a mean (average) of 0.278, a standard deviation of 0.134, a minimum of 0.091, the 25th percentile at 0.200, a median of 0.231, the 75th percentile at 0.333, and a maximum value of 0.857. To ensure all effect labels are displayed in this heatmap, the y-axis presents every second effect label starting with the first, and the x-axis presents every second effect label starting from the second

Associations were identified by using Fisher’s Exact Test across all the specific effects in the entire Binary Matrix. For the 530 observed effects in the Binary Matrix, a total of 193 statistically significant unique, pairwise associations based on Fisher’s Exact Test were observed at *α* ≤ 0.05. All statistically significant pairwise associations are presented in Table S3 of the Supplementary Information and visualized in the heatmap of Fig. 2. Refer to the Section ‘*Association matrix*’ for further details.

#### Biological coherence of associated effects

The consistency of all pairwise effect associations listed in Table S3 of the Supplementary Information was assessed against established scientific understanding. Mechanistic relationships were evaluated in accordance with the published literature, particularly the OECD Guidance Document (GD) 150 (OECD^2018^b). This GD summarizes the investigations in EOGRT studies diagnostic and indicative of endocrine pathways, including estrogen-mediated (both agonistic and antagonistic), androgen-mediated (both agonistic and antagonistic), and steroidogenesis-related activity (refer to Table B.1 in OECD 2018b, page 70).

Associations between identical effects across generations are inherently consistent as they are driven by the same mechanism(s) of toxicity exerted by the substance. This is illustrated by associations, such as post-implantation loss observed in both P0 and P1 generations. Associations involving effects in structurally, anatomically, and functionally related parameters or those with obvious causal relationships—for example, testis and epididymis histopathology; testis or epididymis pathologies and decreased sperm quality; and pre-implantation loss in P0 leading to fewer delivered F1 pups—are logically coherent. This coherence is documented in Table S3 of the Supplementary Information.

The analysis identified statistically significant associations that exemplify causal relationships between associated effects. For instance, decreased numbers of implants directly impact the quantity of delivered pups, highlighting an unambiguous causal link (OECD 2008). Decreased offspring numbers can be traced to diminished sperm motility, emphasizing the male factor’s role in reproductive success (OECD 2008). Decreased sperm quality can directly affect fertilization processes, leading to reduced implantation rates and fewer offspring (Janny and Menezo 1994). Reduced body temperature in sucklings, a consequence of decreased milk intake, emphasizes the importance of maternal provisioning and neonatal capability in early life stages (Henning and Romano 1982). This leads to less milk present in the stomachs of pups, directly correlating with a decline in offspring viability and illustrating the cascade of effects starting from nutritional deficits. Dystocia represents an immediate health concern linked to mortality in both dam and offspring (Baker et al. 2013), and it can be caused by prolonged gestation (Galal et al. 2012). The decrease in implantation rates is linked to structural changes in the endometrium influenced by the complex interplay of endocrine pathways (Timeva et al. 2014). Additionally, a reduction in the number of ovarian follicles may predict decreased female fertility (Jirge 2016).

This analysis shows that all the statistically significant associations can be reconciled with a biological mechanism underpinning the association, and demonstrates that the statistical analysis does not yield associations without a biological underpinning.

### Analyzing response patterns

#### Association matrix

For the identified associations, the ***J*** coefficients were calculated as a measure of the rate of co-occurrence. The resulting symmetrical ***J*** matrix is shown in Fig. 2 and Table S3 of the Supplementary Information lists the ***J*** coefficients.

To provide a brief overview of the Association Matrix, seven association clusters, denoted C1–C7, are visible along the main diagonal in Fig. 2. Clusters C1 and C5 represent associations for reproductive parameters in aF0 and aF1 males, respectively, with the cluster in aF1 exhibiting a higher density of data points. The cluster C2 consists of only two associated effect pairs in pregnant P0 females, whereas no statistically significant associations are observed within P1. C3 and C7 relate to perinatal effects observed in dF1 and dF2 neonates, with several associating effect pairs until weaning in dF1 compared to only two in dF2. Associations regarding female reproductive tissues are visible in cluster C6 in aF1, whereas no such associations are observed in aF0.

Additionally, association clusters extend along the green diagonal in Fig. 2. Effect–effect associations found on or in the vicinity of this line represent identical or different investigations, respectively, across different generations during equivalent life stages, namely aF0 × aF1 (the symbol × stands for across), P0 × P1, and dF1 × dF2. The dense cluster C8 relating to male reproductive parameters in aF0 × aF1 and the less dense cluster C9 for female reproductive parameters are also distinct along the green diagonal, comparable to C1 and C6, respectively. The faint clusters C10 (P0 × P1) and C11 (dF1 × dF2) contain six and four associations, respectively. Remarkably, aF0 × aF1 C9 has no equivalent in P0 × P0 and the P0 × P1 C10 no counterpart in P1 × P1.

Across different generations, life stages, and investigations, numerous pairwise associations were observed, extending beyond the areas around the black and green diagonals. For instance, pre-implantation loss in P0 animals and a decreased number of dF1 pups were associated with 13 and 15 other effects across the heatmap, respectively.

Several significant associations were identified between various parameters within and across generations and life stages, all of which are detailed in Table S3 of the Supplementary Information. Associations with ***J*** coefficients of 0.60 or higher are discussed as examples here. The strongest association was noted for histopathological findings in the epididymis between aF0 and aF1, with a ***J*** coefficient of 0.857 (*p* < 0.001), suggesting a very strong linkage. A similarly significant association for post-natal loss across dF1 and dF2 had a ***J*** coefficient of 0.769 (*p* < 0.001). The causally related association from pre-implantation loss to decreased delivery rate from P1 to dF2 and from P0 to dF1 demonstrated ***J*** coefficients of 0.750 and 0.636 (both *p* < 0.001), respectively. Further, a logical association within the same generation was identified between absence of milk in dF1 pups’ stomachs and reduced body temperature, with a coefficient of 0.667 (*p* < 0.001). Associations between histopathological findings in the epididymis and testis within both aF0 and aF1 were notably strong (***J*** = 0.625). A robust association (***J*** = 0.625) was also seen for testicular histopathology across these generations. These associations are logically coherent, reflecting identical investigations across generations or effects in male reproductive organs that are anatomically and functionally related (for example, testis and epididymis for spermatid and sperm production). Remarkably, an increased anogenital distance in dF2 females correlated significantly with histopathological findings in the epididymis and testis of both aF0 and aF1 (***J*** = 0.600, *p* < 0.01), potentially due to chemical insults on shared endocrine pathways (Table S3 of the Supplementary Information).

#### Generational and effects associations

In the initial step, the dataset of associations was categorized into groups based on (a) occurrences within the same generations versus different generations, as well as (b) for identical effects versus different effects. This categorization was designed to facilitate observation of the dataset’s general behavior, allowing for comparisons of its patterns with respect to generational dependency and the nature of the investigations.

**Fig. 3:**
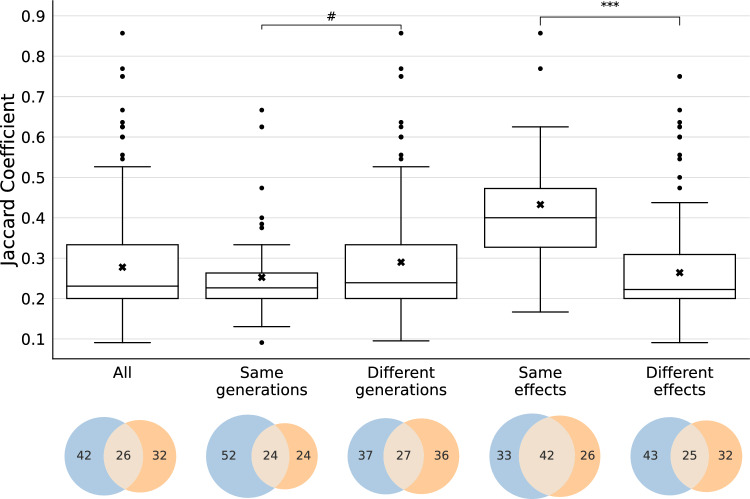
Boxplots and corresponding Venn diagrams illustrate the distribution of Jaccard coefficients (***J***) to show the degree of co-occurrence (DCO). These visualizations span the entire heatmap (“All”), covering comparisons within the same generations (“Same generations”), across different generations (“Different generations”), and for associations involving positive findings in the same or different investigations (“Same effects” and “Different effects”, respectively). The y-axis of the boxplots depicts the ***J*** distribution. Statistical parameters are summarized in Table S4 of the Supplementary Information. The × symbol in the boxplots denotes the mean (average). Comparisons of distributions of ***J*** within the same generations versus across different generations did not reveal any significant differences at a significance level *α* ≤ 0.05 (#, Mann–Whitney *U* test). However, the distributions of correlations for the same versus different effects were found to be significantly different at *α* < 0.001 (***, Mann–Whitney *U* test). These findings indicate that, although there is no significant difference in distributions of ***J*** coefficients between the same and different generations, a significant difference exists between associations of the same and different effects. The Venn diagrams demonstrate the frequency in percent of associated effects occurring in different studies (represented by blue and orange circles) versus within the same studies (indicated by grey overlap) across the categories

The results of this analysis are presented in the boxplots and Venn diagrams of Fig. 3. The boxplots display the distribution of the ***J*** coefficients as a measure of the strength of the association. The graph includes five boxplots: one representing the entire dataset, and others respectively highlighting associations within the same generations, between different generations, involving the same effects, and involving different effects. The Venn diagrams complement these findings by visually representing the % overlap and % single occurrence of associations across these categories, thereby enhancing understanding of the dataset's complexity and the relationships between the different types of associations.

(a) Associations within and across generations

For the entire dataset of statistically significant associations between two investigations which have an effect, the median ***J*** coefficient was 0.231, with a mean of 0.278 ± 0.134. Associations within the same generation had a median ***J*** of 0.226, and the mean was 0.252 ± 0.107. For associations across different generations, the median ***J*** was 0.239, and the mean was 0.290 ± 0.144, as shown in Fig. 3. The Mann–Whitney *U* test did not reveal any significant differences in the ***J*** coefficient distribution between associations within and across generations, at a significance level of *α* ≤ 0.05. The Venn diagrams in Fig. 3 illustrate that the DCO for effects co-occurring within the same generations is 24%, while it is slightly higher, at 27%, across different generations.

Thus, it was concluded that, for all generations in the entire dataset, the co-occurrence of associated effects, whether within the same or across different generations, does not significantly differ, displaying a relatively weak DCO of approximately one-fourth.

(b) Associations for Effects on the Same and Different Investigations

Associations involving an effect on the same investigation demonstrated a median ***J*** coefficient of 0.400 and a mean of 0.433 ± 0.183. In contrast, associations including effects on different investigations exhibited a median ***J*** of 0.222, with a mean of 0.264 ± 0.120, as depicted in Fig. 3. The Mann–Whitney *U* test identified statistically significant differences between the associations of the same effects versus different effects, at a significance level of *α* < 0.001. The Venn diagrams in Fig. 3 show that the DCO for associated same effects is 42%, compared to 25% for associated different effects, indicating that associations for the same effects are 1.7 times more likely to co-occur within a given study than different effects.

For all generations in the entire dataset, this suggests that effects of the same nature significantly co-occur more frequently than those of different natures.

#### The strength of associations between GALS groups is heterogenous

In the second step, the analysis was deepened by examining associations for individual generations, beyond the aggregate view of the entire dataset. This approach aimed to uncover more nuanced patterns of behavior within the data.

**Fig. 4:**
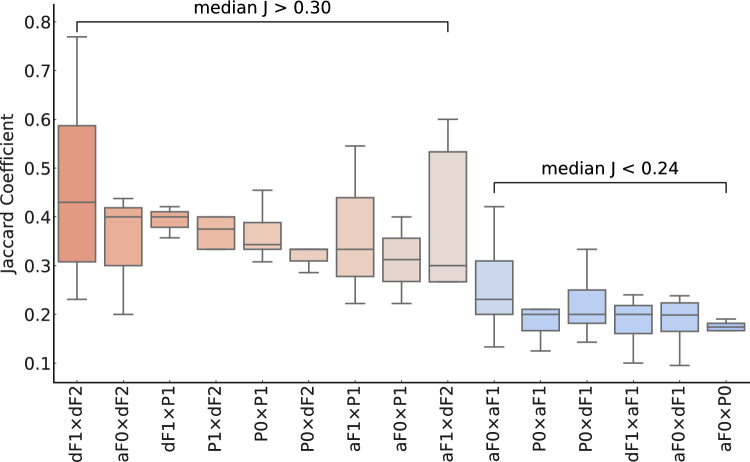
Boxplots of all Jaccard coefficients (***J***) for associations at the animal group level (categories), sorted by descending medians. Upon visual inspection, the categories can be divided into two groups: the first group has associations with a median > 0.30 and the second group with a median < 0.24. Notably, the distinct feature between the first and second group is that the categories with medians > 0.30 encompass associations with effects in P1 and/or dF2. Conversely, the categories with medians < 0.24 comprise all other associations (no associations with P1 or dF2). Statistical parameters are summarized in Table S4 of the Supplementary Information

It was investigated whether the GALS groups had different ***J*** coefficients. Figure 4 presents boxplots of the ***J*** coefficients for associations between investigations categorized into ‘animal groups’, sorted by descending medians. These categories form groups based on the magnitude of their ***J*** coefficients. Specifically, they divided into two distinct groups: one where associations have a median ***J*** coefficient > 0.30, and another with a median ***J*** coefficient < 0.24. A notable difference between these groups is that categories with medians above 0.30 include associations with effects in P1 and/or dF2, whereas categories with medians below 0.24 contain associations with all other generations, excluding those with P1 or dF2.

**Fig. 5:**
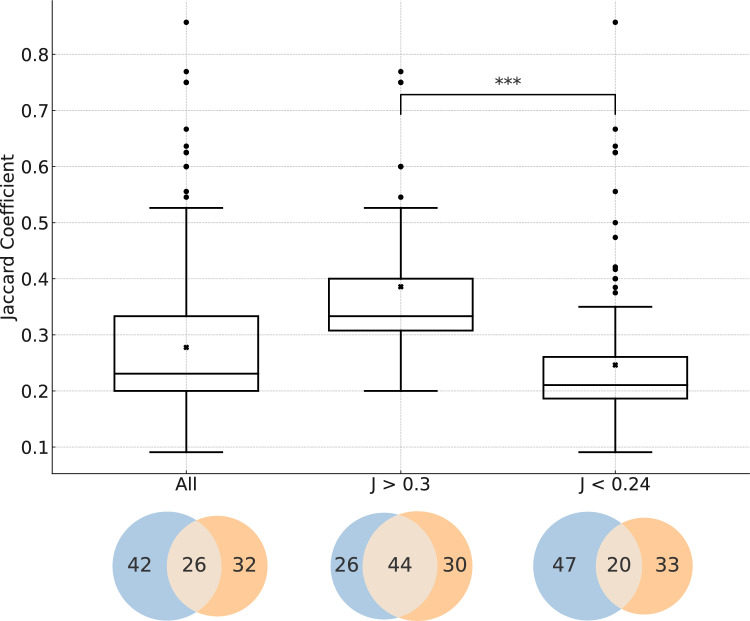
Boxplots and their respective Venn diagrams across the categories: ‘All,’ encompassing the entire set of associations; ‘***J*** > 0.3,’ which includes categories with medians greater than 0.3 (indicating associations across different generations with at least one effect in P1 or dF2); and ‘***J*** < 0.24,’ covering associations with medians less than 0.24 (encompassing all other associations). The Mann–Whitney *U* test, comparing the distribution of ***J*** coefficients between the ‘***J*** > 0.3’ and ‘***J*** < 0.24’ categories, yielded a *p*-value below the significance level *α* of 0.001 (***). This very low *p*-value indicates a significant difference in the distributions of ***J*** coefficients between the two groups, supporting the hypothesis that these groups are distinct in terms of their medians. Specifically, it suggests that the ***J*** coefficients for associations between different generations including effects in P1 and dF2, demonstrate stronger associations compared to all other associations. This implies that associations across different generations including at least one effect in P1 and dF2 co-occur within the same study more frequently than all other associated effects. The Venn diagrams illustrate the percentage of correlated effects occurring in different studies (represented by blue and orange circles) versus those within the same studies (indicated by the overlap) across the three categories. Here, blue and orange denote earlier and later generations, respectively, in the order of aF0, P0, dF1, aF1, P1, and dF2. Statistical parameters for these categories are provided in Table S4 of the Supplementary Information

For further analysis, the categories were aggregated into two groups based on their median ***J*** coefficients—greater than 0.30 and less than 0.24. The results of this comparison are visualized in the boxplots and Venn diagrams in Fig. 5.

A Mann–Whitney *U* test comparing the ***J*** coefficient distributions between these two groups revealed a *p*-value < 0.001, indicating a significant difference in the distributions. The DCO for the group with associations to P1 and/or dF2 was 44%, compared to 20% for the group with associations to all other generations, showcasing a 2.2-fold different DCO.

This comparative analysis concludes that effects associated with P1 and/or dF2 significantly co-occur more frequently within the same study, albeit at a moderate rate of around 44%, than effects associated with other generations, at a low rate of around 20%.

#### Comparing associations of analogous generations and life stages

Building on the previously mentioned findings that the effects associated with P1 and/or dF2 occur significantly more frequently than others, a direct comparison was conducted. This comparison examined the effect associations between (a) P0 × dF1 and P1 × dF2, (b) aF0 × P0 and aF1 × P1, and (c) aF0 × aF1, P0 × P1, and dF1 × dF2. The objective is to determine if there is a biologically plausible connection for association between GALS groups.

(a) Associations between effects measured in parents and their offspring

**Fig. 6:**
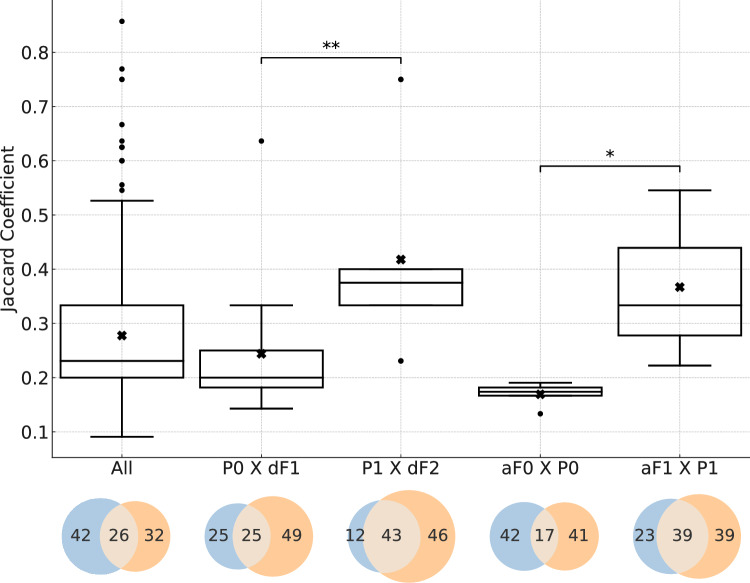
Distribution of Jaccard (***J***) coefficients displaying the associations between effects in equivalent animal groups across different generations, specifically between P0 × dF1 and P1 × dF2, as well as aF0 × P0 and aF1 × P1. The strength of association, measured by the degree overlap, which is the co-occurrence of associated effects within the same studies compared to their occurrence across all studies, are higher for associations that include P1 and dF2 effects. The degree of co-occurrence (overlap) based on the number of associated effects for P0 × dF1 is 25%, for P1 × dF2 43%, for aF0 × P0 17%, and aF1 × P1 39%, as shown in the respective Venn diagrams. The distribution of ***J*** coefficients is significantly differed between P0 × dF1 and P1 × dF2, as well as between aF0 × P0 and aF1 × P1, with a significance level of *p* ≤ 0.01 (**) and *p* ≤ 0.05 (*), respectively, using the Mann–Whitney *U* test. The boxplot for all associations is given as reference. Statistical parameters for these categories are provided in Table S4 of the Supplementary Information

Associations for P0 × dF1 displayed a median ***J*** coefficient of 0.200 and a mean of 0.244 ± 0.128. The associations for the equivalent generations P1 × dF2 had a median of 0.375, and the mean was 0.418 ± 0.185 (Fig. 6). The distribution of ***J*** coefficients significantly differed between P0 × dF1 and P1 × dF2, with a significance level *α* < 0.01, according to the Mann–Whitney *U* test. The Venn diagrams in Fig. 6 illustrate that the DCO for P0 × dF1 associations is 25%, whereas it is 44% for P1 × dF2 associations, indicating approximately a 1.8-fold difference. Based on this comparison of associations in equivalent generations, it is concluded that associated effects co-occur significantly more frequently in P1 × dF2 compared to P0 × dF1 in the same study.

(b) Associations between effects measured in adults and the corresponding parents

Associations for aF0 × P0 displayed a median ***J*** coefficient of 0.174 and a mean of 0.169 ± 0.022. The associations for the equivalent generations aF1 × P1 had a median of 0.333, and the mean was 0.367 ± 0.164 (Fig. 6). The distribution of ***J*** coefficients significantly differed between aF0 × P0 and aF1 × P1, with a significance level of *p* ≤ 0.05, using the Mann–Whitney *U* test. The Venn diagrams in Fig. 6 illustrate that the DCO for aF0 × P0 associations is 17%, and for aF1 × P1 associations, it is 39%, meaning it is approximately 2.3-fold higher for aF1 × P1. Based on this comparison of associations, it is concluded that associated effects co-occur significantly more frequently in the same study for aF1 × P1 compared to the equivalent generation aF0 × P0.

(c) Associations between effects measured in different generations for adult, parental, and offspring investigations

**Fig. 7:**
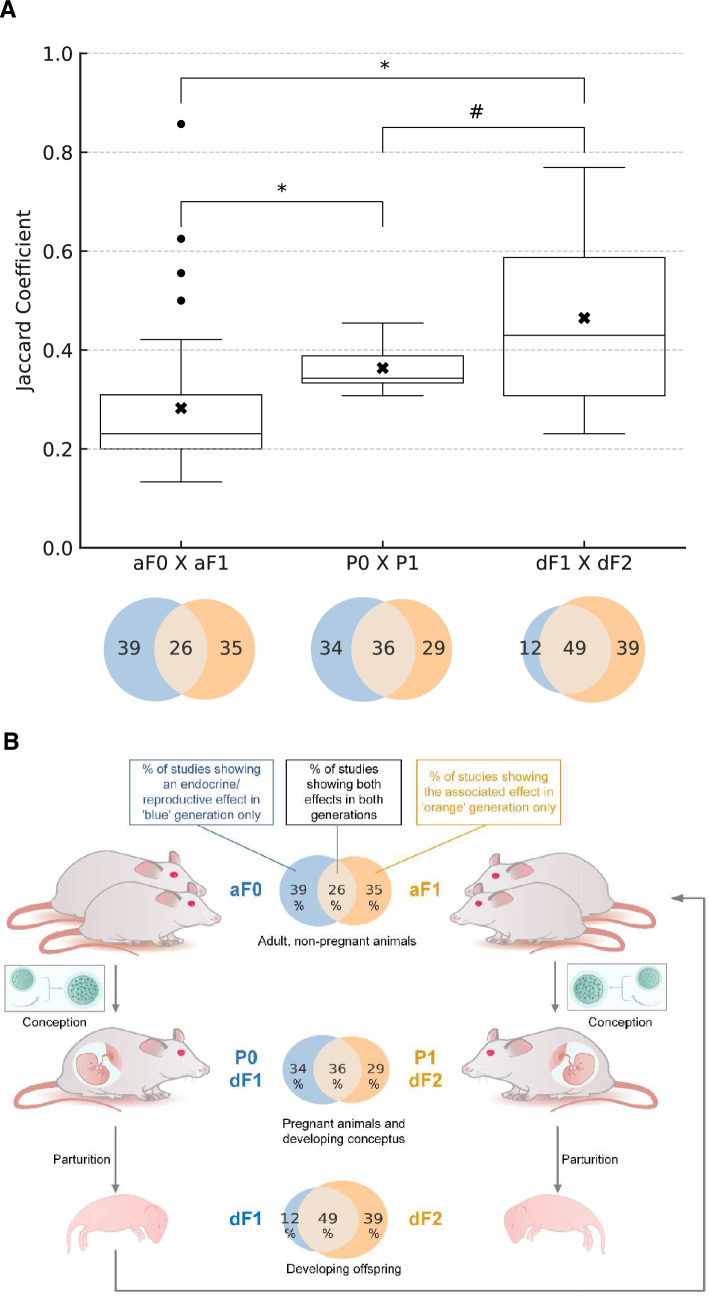
Panel **A**: Distribution of Jaccard (***J***) coefficients displaying the associations between effects in consecutive equivalent animal groups across different generations, specifically between aF0 and aF1 (aF0 × aF1), P0 and P1 (P0 × P1), and dF1 and dF2 (dF1 × dF2). This analysis corresponds to the associations displayed on and around the green diagonal in the heatmap shown in Fig. 2. Like the previous results, the strength of association is higher for associations that include P1 and dF2 effects. The degree of co-occurrence based on the number of associated effects for aF0 × aF1 is 26%, for P0 × P1 36%, and for dF1 × dF2 49%, as can be seen from the respective Venn diagrams. The distribution of ***J*** coefficients significantly differed between aF0 × aF1 and P0 × P1, as well as between aF0 × aF1 and dF1 × dF2, with a significance level of *p* ≤ 0.05 (*, using the Mann–Whitney *U* test). Statistical parameters for these categories are provided in Table S4 of the Supplementary Information. There is no statistically significant different distribution of ***J*** between P0 × P1 and dF1 × dF2 (#, using the Mann/Whitney *U* test). Panel **B**: Illustrating the occurrence of associated effects in the rat life cycle of the EOGRT study

Associations for the consecutive equivalent life stages and generations aF0 × aF1, P0 × P1, and dF1 × dF2 exhibited median ***J*** coefficients of 0.231, 0.343, and 0.430, with means of 0.282 ± 0.140, 0.364 ± 0.054, and 0.465 ± 0.237, respectively (Fig. 7A). These three associations correspond to data points displayed in the sections intersected by the green diagonal in the heatmap shown in Fig. 2. Like the previous results, the strength of associations is higher for associations that include P1 and/or dF2 effects. The distribution of ***J*** coefficients significantly differed between aF0 × aF1 and P0 × P1, as well as between aF0 × aF1 and dF1 × dF2, with *p *≤ 0.05 in the Mann–Whitney *U* test. However, no significant difference was observed between P0 × P1 and dF1 × dF2, suggesting that P1 and dF2 may enhance the strength of association in a comparable manner. The Venn diagrams in Fig. 7A and B show that the DCO for aF0 × aF1 associations is 26%, compared to 36% for P0 × P1 and 49% for dF1 × dF2, which are approximately 1.4 and 1.9 times higher compared to aF0 × aF1, respectively. Based on this comparison of associations across consecutive equivalent life stages and generations, it is concluded that associated effects occur significantly more frequently together in the same study for associations involving P1 and/or dF2 effects.

### Summary of results

The binary matrix contains 530 effects, among which 193 pairwise associations have been identified at a significance level of *α* ≤ 0.05 (Fig. 2). Across the entire matrix, the strength of these associations is relatively low, with a ***J*** coefficient of approximately 0.23 and a DCO of 26%. This analysis reveals no statistical difference between associations within the same generations and those across different generations. However, median ***J*** coefficients are statistically significantly higher for associations involving the effects in the same investigations (median ***J*** = 0.400, DCO = 42%) compared to those involving effects in different investigations (median ***J*** = 0.222, DCO = 25%) as illustrated in Fig. 3.

Further analysis of the data reveals that associations involving dF2 and/or P1 exhibit statistically significantly higher median ***J*** coefficients, ranging from 0.300 to 0.430, in contrast to all other associations, which range from 0.182 to 0.231 (Fig. 4). Consequently, two distinct groups of associations have been identified: one with median ***J*** coefficients > 0.30 and another with coefficients < 0.24. Notably, all associations with median ***J*** coefficients greater than 0.30 involve effects observed in P1 and/or dF2: the DCO for associations involving P1 and/or dF2 is approximately 44%, compared to 20% for all other associations (Fig. 5).

An analysis of equivalent generations and life stages yields similar findings: associations involving P1 and/or dF2 are associated with statistically higher strengths compared to those excluding P1 and/or dF2, with median ***J*** coefficients for P1 × dF2 (0.375) and aF1 × P1 (0.333) being higher than those for P0 × dF1 (0.200) and aF0 × P0 (0.174), respectively. Moreover, median ***J*** coefficients increase in the following order of consecutive equivalent associations: aF0 × aF1 (0.231), P0 × P1 (0.343), and dF1 × dF2 (0.430) again supporting the finding that associations with effects observed in P1 and dF2 are stronger than others.

This analysis uncovers a consistent pattern: associations across generations involving effects observed in P1 and/or dF2 are significantly stronger and exhibit higher DCOs compared to associations among other generations, indicating intergenerational differences in response to endocrine and reproductive challenges. Associations for aF0, P0, dF1, and aF1 demonstrate low median co-occurrence rates of approximately 17 to 25%, whereas associations with P1 and/or dF2 show moderate median rates of around 40 to 44%.

## Discussion

This analysis of endocrine and reproductive effects in EOGRT studies, as depicted in Fig. 1 and covering a structurally diverse set of 112 substances (detailed in Table S1 of the Supplementary Information), has yielded several critical insights into generational effects of toxicological responses.

First, the analysis confirmed the reliability and adequacy of the dataset for producing meaningful results. Through the creation of a dense binary matrix featuring 530 effects (as shown in Fig. S1 of the Supplementary Information), 193 statistically significant associations for pairwise effects were identified across all generations and life stages (see Fig. 2). Importantly, the biological coherence of the data was confirmed (refer to Table S3 of the Supplementary Information).

The investigation into general response patterns both within and across generations revealed that the co-occurrence of associated effects is relatively infrequent. This observation is supported by associations that exhibit low median ***J*** coefficients of 0.233 and a weak DCO of 26% across the entire association matrix. Notably, 50% of the identified associations had a DCO ranging from 9 to 26% within the same study (Table S4 of the Supplementary Information).

Remarkably, in 80% of EOGRT studies reporting positive findings, relevance of these effects was disputed in the Substance Data Sheet. The justification was that the effects appeared isolated to a single life stage/generation and not observed in subsequent ones, suggesting that an effect is considered real only if confirmed in later life stages/generations, the so-called continuity assumption.

However, the findings of this article challenge this traditional expectation of continuity if an effect was indeed related to the treatment. Instead, this analysis reveals that the simultaneous occurrence of treatment-related endocrine and reproductive effects across different generations and life stages within the same study is rather low.

When analyzing the response patterns for P1 and dF2, it was found that the associations involving these animal groups exhibited higher strengths (meaning higher ***J*** coefficients) and DCOs compared to those involving other animal groups. This pattern was consistently observed in various comparative analyses: across associations for individual generations (Fig. 4), as well as by comparing equivalent generations (Fig. 5) and consecutive equivalent life stages and generations (Fig. 6). For example, increased anogenital distance in dF2 females shows an average ***J*** coefficient of 0.444 (standard deviation = 1.20), whereas in dF1, the average ***J*** coefficient is 0.228 (standard deviation = 0.0740). Similarly, pre-implantation loss in P1 shows an average ***J*** coefficient of 0.447 (standard deviation = 0.174), whereas in P0, the average ***J*** coefficient is 0.237 (standard deviation = 0.132) (Table S3 of the Supplementary Information).

The question, therefore, is what could be the reasons for the differences in strengths and DCOs associated with P1 and/or dF2.

**Fig. 8:**
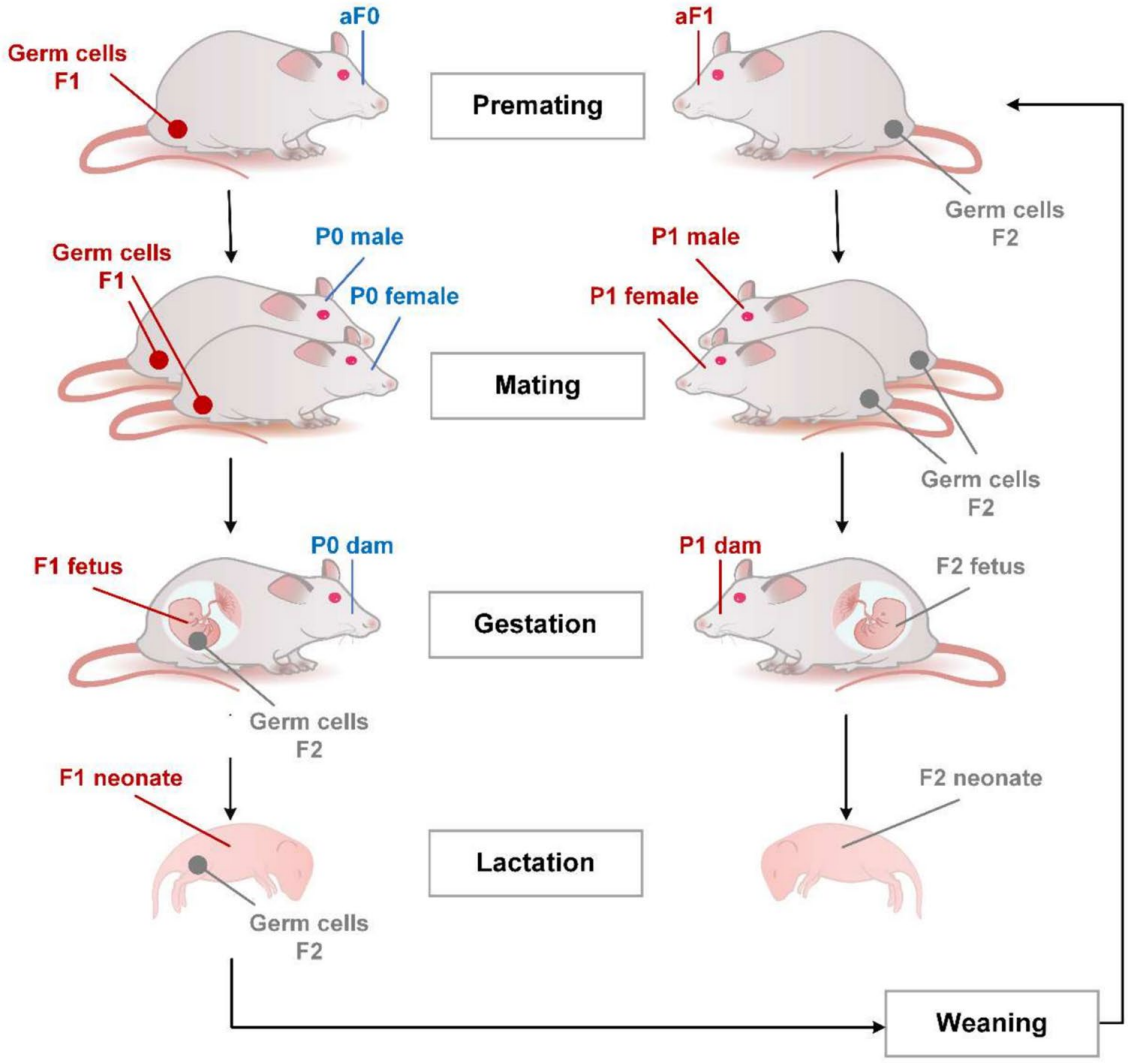
Differences in exposure duration in the EOGRT study: Exposure to F1 (red) commences with the dosing of young aF0 animals (blue) at the initiation of the EOGRT study, owing to the exposure of their germ cells, which subsequently produce F1 (referred to as ‘Germ cells F1’). Conversely, exposure to F2 (grey) begins with the dosing of pregnant P0 females (dams), through the germ cells in the F1 fetus that leads to the development of F2 (referred to as ‘Germ cells F2’). This different exposure duration might explain the higher strength of associations involving dF2 effects. The higher strength of associations for P1 might be explained by the influence of the F2 conceptus on the P1 dam

In this context, it is important to recognize that the exposure durations for dF1 and dF2 are different in the EOGRT study: The development of dF2 is already influenced by the in utero exposure of the germ cells of the dF1 conceptus—the germ cells that will then produce the F2 generation—during the pregnancy of P0. On the other hand, the exposure of F1 begins only with the initiation of dosing in young P0 animals in the EOGRT study, affecting its germ cells that will then produce the dF1 animals (Fig. 8).

Therefore, the observed inter-generational differences might be explained by different exposure durations, which also cover different life stages and sensitivity windows. These differences could lead to varied genetic and epigenetic modifications in dF1 compared to dF2, in turn causing distinct alterations in homeostasis and metabolism that shape toxicological responses (Breton et al. 2021; Zheng et al. 2023; Rattan and Flaws 2019; Chianese et al. 2018). Such factors might contribute to a complex interplay of exposure-induced changes that influence an organism's susceptibility to toxic effects.

Remarkably, associations for P1 effects also show higher strengths (higher ***J*** coefficients) and DCOs compared to aF1, even though the aF1 and P1 animals are identical animals of the same F1 generation but belong to different life stages. This striking dissimilarity among animals of the same generation might be explained, at least in part, by the differences in physiological states, such as gestation, delivery, and lactation of P1 females, compared to the non-mated, non-pregnant aF1.

The dF2 generation also influences the P1 pregnancy and its outcome. This influence is due to the interaction between the dF2 conceptus and its pregnant mother (P1), encompassing maternal–fetal interactions or maternal–fetal cross-talk (Murphy et al. 2006; Garcia-Flores et al. 2024). In other words, chemical insults on the development of the dF2 fetus during the prolonged exposure duration, compared to dF1, might alter the P1-dF2 cross-talk compared to that of P0-dF1, resulting in different responses to chemical insults. Supporting this hypothesis is the fact that 92% (23 out of 25) of the associations involving P1 are related to effects significantly governed by the cross-talk between the P1 dam and the dF2 conceptus, including pre-implantation loss, post-implantation loss, increased gestation length (indicating delayed birth), and dystocia (problems during delivery) (Table S3 of the Supplementary Information). The remaining 8% (2 out of 25) of associations involving P1 are related to a decreased fertility index, which might result from a longer exposure duration to the germ cells, as explained above (Table S3 of the Supplementary Information).

Taken together, the findings in this report lead to a critical reassessment of the hypotheses, which posited: (1) a high level of co-occurrence of associated treatment-related effects across different life stages and generations, and (2) that there are no significant differences in the strength of these associations. Based on the presented findings, both hypotheses are rejected because (1) only low to moderate median ***J*** coefficients and DCOs have been observed, and (2) significant differences in the strengths of associations exist, particularly between those involving P1 and/or dF2 and those that do not.

The presented analysis advocates for recognizing animals of different generations and life stages as distinct entities, emphasizing that confirmation of findings across generations and life stages should not be regarded as a default approach but rather considered complementary. This perspective is crucial for accurately interpreting toxicological data, highlighting the importance of analyzing each observed effect independently within its generational context.

In conclusion, the findings of this article advocate for a nuanced interpretation of generational effects in toxicological evaluations of endocrine and reproductive effects.

## Conclusion

Confirmation of treatment-related reproductive and endocrine effects across different generations and life stages within the same study is relatively infrequent. It is much more common for animals of different generations and life stages to exhibit varied endocrine and reproductive responses. Therefore, positive findings observed exclusively in one generation and/or life stage and not in others should not be dismissed due to the absence of continuity. Instead, they should be regarded as complementary and evaluated on their own merits.

## Supplementary Information

Below is the link to the electronic supplementary material.

Supplementary file1 (PDF 463 KB)

## Abbreviations

α: Significance level
aF0: Adult initial generation
aF1: Adult first filial generation
dF1: Developing first filial generation
dF2: Developing second filial generation
EOGRT: Extended one-generation reproductive toxicity
F1: First filial generation
F2: Second filial generation
GALS: Generation and life stage
ICH: International council for harmonisation of technical requirements for pharmaceuticals for human use
IUCLID: International uniform chemical information database
***J***: Jaccard coefficient
P0: Parental animals of the adult initial generation
P1: Parental animals of the adult first generation
REACH: Registration, evaluation, authorisation and restriction of chemicals
TG: Test guideline
WHO/IPCS: World Health Organization/International Programme on Chemical Safety

## Footnotes

1 The Substance Data Sheets can be accessed via the 'Search for chemicals' function on the ECHA website at https://echa.europa.eu/information-on-chemicals.
