# Supplementary material for: Differences in endocrine and reproductive responses to substance exposure across generations: highlighting the importance of complementary findings
Source: Arch Toxicol. 2024 Jul 18;98(10):3215–30. doi: 10.1007/s00204-024-03813-3 (PMC11402854; doi:10.1007/s00204-024-03813-3)
Supplement: Supplementary file 1 — Supplementary file1 (PDF 463 KB) [file 204_2024_3813_MOESM1_ESM.pdf]

Supplementary Information

Title

Differences in Endocrine and Reproductive Responses to Substance Exposure Across Generations: Highlighting the Importance of Complementary Findings

Author

Ingo Bichlmaier

Affiliation

European Chemicals Agency, Hazard Assessment Directorate, Telakkakatu 6, 00150 Helsinki, Finland

Figure S1. Distribution of the 530 observed effects across the 85 investigations (x-axis) and 112 EOGRT studies (y-axis). The data points in the matrix represent observed (positive) findings.

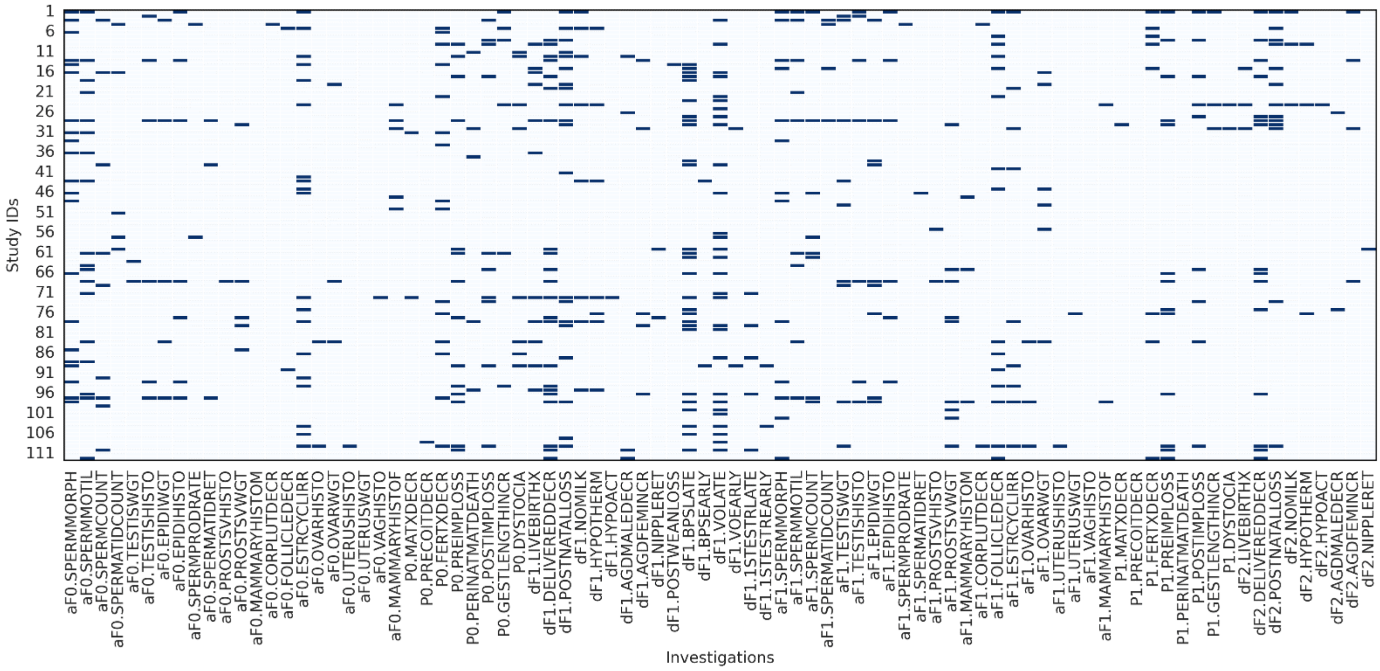

Figure S2. Density of observed effects in the Binary Matrix. Frequency of positive investigations and co-occurrence with other positive findings in 112 EOGRT studies. **A**: The co-occurrence of the 530 observed effects is analyzed across the generations of adult, parental, and developing animal groups (aF0 and aF1, P0 and P1, dF1 and dF2, respectively). Each finding occurred on average (mean) in 27 studies per GALS group with a standard deviation of ca. 13. **B**: The boxplot summarizes the frequency of occurrences of all effects in the Binary Matrix within and across generations: minimum at 14, 25th Percentile at 16, median at ca. 23, 75th percentile at 33 and maximum at 63 studies.

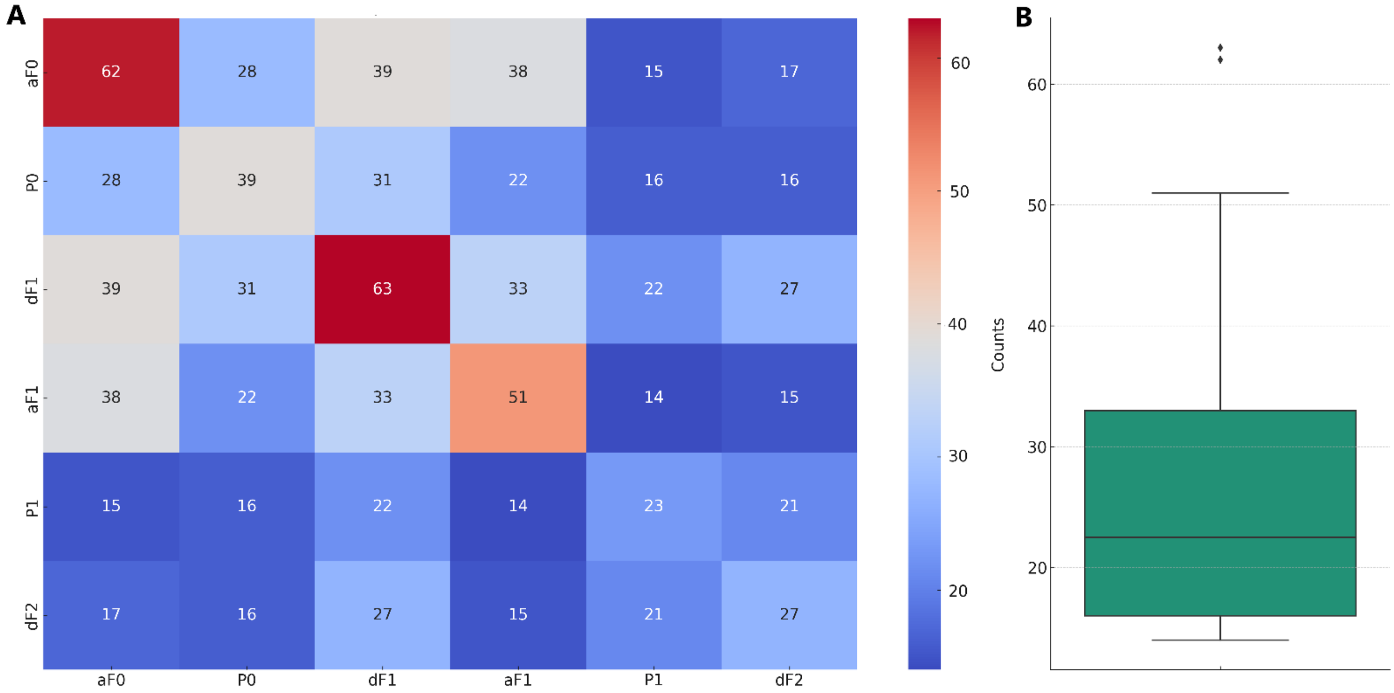

Table S1. 112 substances were used as test items in the EOGRT studies. Identifiers, such as EC numbers and CAS registration numbers, are provided. The column labeled "F2" indicates whether the F2 generation was produced ('y' for yes) or not ('n' for no).

Substance name	EC number	CAS number	F2	Study finalization
Tris[2-[2-(2-methoxyethoxy)ethoxy]ethyl] orthoborate	*250-418-4*	*30989-05-0*	*y*	*2022*
Tris(2-ethylhexyl) phosphate	*201-116-6*	*78-42-2*	*n*	*2020*
Trifluoroacetic acid	*200-929-3*	*76-05-1*	*n*	*2022*
tert-Dodecanethiol	*246-619-1*	*25103-58-6*	*y*	*2021*
tert-butyl 2-ethylperoxyhexanoate	*221-110-7*	*3006-82-4*	*y*	*2020*
Sulphamidic acid	*226-218-8*	*5329-14-6*	*n*	*2021*
Reaction mass of dimethyl adipate and dimethyl glutarate and dimethyl succinate	*906-170-0*	*NA*	*y*	*2021*
Propylidynetrimethanol	*201-074-9*	*77-99-6*	*y*	*2020*
Octene, hydroformylation products, low-boiling	273-110-1	68938-03-4	*y*	*2021*
Octene, hydroformylation products, high-boiling	271-237-7	68526-89-6	*n*	*2021*
N-1,3-dimethylbutyl-N'-phenyl-p-phenylenediamine	*212-344-0*	*793-24-8*	*n*	*2019*
Mequinol	*205-769-8*	*150-76-5*	*n*	*2019*
Melamine	*203-615-4*	*108-78-1*	*y*	*2020*
Hexafluoropropene	*204-127-4*	*116-15-4*	*n*	*2019*
Geraniol	*203-377-1*	*106-24-1*	*y*	*2021*
Ethanol, 2,2'-iminobis-, N-C12-18-alkyl derivs	*276-014-8*	*71786-60-2*	*n*	*2023*
Benzenamine, N-phenyl-, reaction products with 2,4,4-trimethylpentene	*270-128-1*	*68411-46-1*	*y*	*2021*
2-Butyne-1,4-diol, polymer with 2-(chloromethyl)oxirane, brominated, dehydrochlorinated, methoxylated	*614-503-3*	*68441-62-3*	*n*	*2020*
2-Methylpentane-2,4-diol	*203-489-0*	*107-41-5*	*y*	*2021*
2-Amino-2-methylpropanol	*204-709-8*	*124-68-5*	*n*	*2021*
2,4,6-Trinitrotoluene	*204-289-6*	*118-96-7*	*n*	*2019*
2,4,6-trichloro-1,3,5-triazine	*203-614-9*	*108-77-0*	*n*	*2019*
2,2'-ethylenedioxydiethyl bis(2-ethylhexanoate)	*202-319-2*	*94-28-0*	*y*	*2019*
1,3-diphenylguanidine	*203-002-1*	*102-06-7*	*y*	*2021*
Propylidynetrimethyl trimethacrylate	*221-950-4*	*3290-92-4*	*y*	*2022*
Methyl 4-hydroxybenzoate	*202-785-7*	*99-76-3*	*y*	*2021*
Dioctyltin oxide	*212-791-1*	*870-08-6*	*y*	*2020*
Dapsone	*201-248-4*	*80-08-0*	*y*	*2020*
3,5-Dimethylpyrazole	*200-657-5*	*67-51-6*	*y*	*2019*
1,3-dihydro-4(or 5)-methyl-2H-benzimidazole-2-thione, zinc salt	*262-872-0*	*61617-00-3*	*y*	*2019*
1,1,3,3-tetramethylbutyl peroxyneodecanoate	*257-077-0*	*51240-95-0*	*n*	*2022*
vinyl neononanoate	*259-160-7*	*54423-67-5*	*n*	*2022*
Trimethoxyvinylsilane	*220-449-8*	*2768-02-7*	*y*	*2021*

Magnesium, bis[2-(hydroxy-.kappa.O)benzoato-.kappa.O]-, ar, ar'-di-C14-18-alkyl derivs.	*931-371-5*	*NA*	*y*	*2021*
Propyl acetate	*203-686-1*	*109-60-4*	*y*	*2022*
Isopentyl acetate	*204-662-3*	*123-92-2*	*n*	*2019*
Cyclohex-1,4-ylenedimethanol	*203-268-9*	*105-08-8*	*y*	*2019*
Citral	*226-394-6*	*5392-40-5*	*y*	*2021*
2,5-Furandione, dihydro-, mono-C15-20-alkenyl derivs.	*272-221-2*	*68784-12-3*	*n*	*2021*
2,2-bis[[(1-oxopentyl)oxy]methyl]propane-1,3-diyl divalerate	*239-937-7*	*15834-04-5*	*n*	*2022*
2-Dimethylaminoethanol	203-542-8	108-01-0	*n*	*2021*
Poly[oxy(methyl-1,2-ethanediyl)], alpha.-(2-aminomethylethyl)-.omega.-(2-aminomethylethoxy)-	618-561-0	NA	*n*	*2021*
2-Butanone, peroxide	700-954-4	1338-23-4	*n*	*2021*
Reaction mass of 2-methylbutyl acetate and pentyl acetate	908-918-1	NA	*n*	*2020*
reaction mass of 1-(1,2,3,4,5,6,7,8-octahydro-2,3,8,8-tetramethyl-2-naphthyl)ethan-1-one and 1-(1,2,3,4,6,7,8,8a-octahydro-2,3,8,8-tetramethyl-2-naphthyl)ethan-1-one and 1-(1,2,3,5,6,7,8,8a-octahydro-2,3,8,8-tetramethyl-2-naphthyl)ethan-1-one	915-730-3	NA	*n*	*2020*
Pentaerythritol	204-104-9	115-77-5	*n*	*2021*
N,N''-(isobutylidene)diurea	228-055-8	6104-30-9	*n*	*2022*
Hexahydro-1,3,5-trimethyl-1,3,5-triazine	203-612-8	108-74-7	*n*	*2020*
Ethanol, 2,2'-oxybis-, reaction products with ammonia, morpholine derivs. residues	272-712-1	68909-77-3	*n*	*2021*
Dimethylamine	204-697-4	124-40-3	*y*	*2023*
Dichloromethylbenzene	249-854-8	29797-40-8	*n*	*2020*
Sodium polysulfide aluminosilicate with a SOD-type framework structure	701-340-9	NA	*n*	*2022*
Barium chloride	233-788-1	10361-37-2	*n*	*2020*
Ethanaminium, 2-hydroxy-N-(2-hydroxyethyl)-N,N-dimethyl-, esters with C16-18 and C18-unsatd. fatty acids, chlorides	620-174-7	1079184-43-2	*n*	*2020*
5-methylhexan-2-one	203-737-8	110-12-3	*y*	*2019*
3-Methylbutan-1-ol	204-633-5	123-51-3	*n*	*2020*
2-Pyrrolidone	210-483-1	616-45-5	*n*	*2020*
2,2'-[oxybis(methylene)]bis[2-ethylpropane-1,3-diol]	245-509-0	23235-61-2	*n*	*2020*
1-Hexanol, 2-ethyl-, manuf. of, by-products from, distn. residues	271-832-1	68609-68-7	*n*	*2020*
Menthol	*201-939-0*	*89-78-1*	*y*	*2020*
Chloroethane	200-830-5	75-00-3	*n*	*2020*

2,2'-(octadec-9-enylimino)bisethanol	246-807-3<br>233-520-3	25307-17-9	*n*	*2021*
Phosphinic acid, P,P-diethyl-, aluminum salt (3:1)	428-310-5	225789-38-8	*n*	*2020*
Reaction mass of bis(oxiran-2-ylmethyl) terephthalate and tris(oxiran-2-ylmethyl) benzene-1,2,4-tricarboxylate	940-592-6	NA	*n*	*2020*
4,4'-sulphonyldiphenol	201-250-5	80-09-1	*y*	*2019*
2-(4-tert-butylbenzyl)propionaldehyde	201-289-8	80-54-6	*y*	*2017*
1,3-diethyldiphenylurea	201-645-2	85-98-3	*n*	*2020*
2,4,6-tris(dimethylaminomethyl)phenol	202-013-9	90-72-2	*y*	*2020*
Biphenyl	202-163-5	92-52-4	*y*	*2018*
Propyl 4-hydroxybenzoate	202-307-7	94-13-3	*n*	*2021*
4-tert-butylpyrocatechol	202-653-9	98-29-3	*n*	*2021*
Acetophenone	202-708-7	98-86-2	*n*	*2021*
4-hydroxy-4-methylpentan-2-one	204-626-7	123-42-2	*y*	*2020*
2-(2-aminoethoxy)ethanol	213-195-4	929-06-6	*n*	*2021*
1,3,4,6,7,8-hexahydro-4,6,6,7,8,8-hexamethylindeno[5,6-c]pyran	214-946-9	1222-05-5	*y*	*2021*
1-(5,6,7,8-tetrahydro-3,5,5,6,8,8-hexamethyl-2-naphthyl)ethan-1-one	216-133-4	1506-02-1	*y*	*2020*
2,2'-dimethyl-4,4'-methylenebis(cyclohexylamine)	229-962-1	6864-37-5	*n*	*2020*
Diammonium peroxodisulphate	231-786-5	7727-54-0	*n*	*2021*
Zinc bis(diethyldithiocarbamate)	238-270-9	14324-55-1	*n*	*2021*
N,N-dimethyldecan-1-amide	238-405-1	14433-76-2	*y*	*2021*
Sodium 4-propoxycarbonylphenoxide	252-488-1	35285-69-9	*n*	*2021*
(1-methyl-1,2-ethanediyl)bis[oxy(methyl-2,1-ethanediyl)] diacrylate	256-032-2	42978-66-5	*n*	*2019*
Phenol, isopropylated, phosphate (3:1)	273-066-3	68937-41-7	*y*	*2021*
2-Oxetanone, 3-C14-16-alkyl 4-C15-17-alkylidene derivs.	308-760-8	98246-87-8	*n*	*2021*
A mixture of: cis-tetrahydro-2-isobutyl-4-methylpyran-4-ol; trans-tetrahydro-2-isobutyl-4-methylpyran-4-ol	405-040-6	63500-71-0	*n*	*2018*
4,4'-Isopropylidenediphenol, propoxylated	500-097-4	37353-75-6	*y*	*2018*
4,4'-Isopropylidenediphenol, oligomeric reaction products with 1-chloro-2,3-epoxypropane, esters with acrylic acid	500-130-2	55818-57-0	*n*	*2020*
Oligomerisation and alkylation reaction products of 2-phenylpropene and phenol	700-960-7	NA	*n*	*2019*
Reaction mass of diisopropyl-1,1'-biphenyl and tris(1-methylethyl)-1,1'-biphenyl	915-589-8	NA	*y*	*2020*

Reaction mass of 2-(1,1-dimethylpropyl)anthraquinone and 2-(1,2-dimethylpropyl)anthraquinone	915-623-1	NA	*n*	*2021*
Fatty acids, C16-18 even numbered, reaction products with triethanolamine, di-Me sulfate-quaternized	931-209-3	NA	*n*	*2020*
3-C12-18-(even numbered)-alkylamido-N,N-dimethylpropan-1-amino oxide	939-581-9	NA	*n*	*2021*
Morpholine	203-815-1	110-91-8	*n*	*2021*
2-ethylhexanoic acid	205-743-6	149-57-5	*y*	*2016*
3,5,5-trimethylhexanoic acid	221-975-0	3302-10-1	*n*	*2019*
Octocrilene	228-250-8	6197-30-4	*y*	*2019*
4-methylmorpholine 4-oxide, monohydrate	231-391-8	7529-22-8	*n*	*2020*
2,2'-iminodiethanol	203-868-0	111-42-2	*n*	*2018*
Butyl acrylate	205-480-7	141-32-2	*n*	*2017*
3,7-dimethyloctan-3-ol	201-133-9	78-69-3	*n*	*2022*
Acrylic acid, monoester with propane-1,2-diol	247-118-0	25584-83-2	*n*	*2023*
Zinc bis(dibenzyldithiocarbamate)	238-778-0	14726-36-4	*n*	*2022*
3,7-dimethyloct-6-enenitrile	257-288-8	51566-62-2	*n*	*2021*
3-aminopropyldiethylamine	203-236-4	104-78-9	*y*	*2021*
Polysulfides, bis[3-(triethoxysilyl)propyl]	915-673-4	NA	*n*	*2022*
[1,3(or 1,4)-phenylenebis(1-methylethylidene)]bis[tert-butyl] peroxide	246-678-3	25155-25-3	*n*	*2022*
Isononanoic acid, C16-18 (even numbered)-alkyl esters	601-141-6	111937-03-2	*n*	*2022*
1,2,3,4-tetrahydronaphthalene	204-340-2	119-64-2	*n*	*2021*
Decahydronaphthalene	202-046-9	91-17-8	*y*	*2022*
Bis(2,2,6,6-tetramethyl-4-piperidyl) sebacate	258-207-9	52829-07-9	*y*	*2020*
Methyl 2-naphthyl ether	202-213-6	93-04-9	*y*	*2020*
Carbon disulphide	200-843-6	75-15-0	*n*	*2019*
2-(4-methylcyclohex-3-en-1-yl)propan-2-ol	701-188-3	NA	*y*	*2021*

Table S2. The following 46 endpoints related to endocrine activity and male and female reproduction, mating, gestation, parturition, lactation, weaning, anogenital distance, nipple/areola retention, and sexual maturation were evaluated. The abbreviations used in other graphs in this paper are shown in brackets.

Male reproductive parameters investigated in aF0 and aF1<br>• Abnormal sperm morphology (SPERMMORPH)<br>• Decreased sperm motility (SPERMMOTIL)<br>• Decreased epididymal sperm counts (SPERMCOUNT)<br>• Decreased testicular spermatid counts (SPERMATIDCOUNT)<br>• Changes in absolute testis weights/ size (TESTISWGT)<br>• Testis histopathology (TESTISHISTO)<br>• Changes in absolute epididymis weights/ size (EPIDIWGT)<br>• Epididymis histopathology (EPIDIHISTO)<br>• Decreased sperm production rate (SPERMPRODRATE)<br>• Spermatid retention (SPERMATIDRET)<br>• Prostate/ seminal vesicles histopathology (PROSTSVHISTO)<br>• Prostate/ seminal vesicles weights/ size (PROSTSVWGT)<br>• Mammary gland histopathology in males (MAMMARYHISTOM)	***Female reproductive parameters in aF0 and aF1***<br>• Decreased corpora lutea counts (CORPLUTDECR)<br>• Decreased ovarian follicle counts (FOLLICLEDECR)<br>• Irregular estrus cycling (ESTRCYCLIRR)<br>• Ovaries histopathology (OVARHISTO)<br>• Changes in absolute ovary/ oviducts weights (OVARWGT)<br>• Uterus histopathology (UTERUSHISTO)<br>• Changes in absolute uterus weights (UTERUSWGT)<br>• Vagina histopathology (VAGHISTO)<br>• Mammary gland histopathology in females (MAMMARYHISTOF)
Mating investigated in P0 and P1<br>• Decreased mating index (MATXDECR)<br>• Increased precoital interval (PRECOITINCR)<br>• Decreased fertility index (FERTXDECR)	***Gestation and parturition investigated in P0 and P1***<br>• Increased pre-implantation loss (PREIMPLOSS)<br>• Peri-natal maternal death (PERINATMATDEATH)<br>• Increased post-implantation loss (POSTIMPLOSS)<br>• Increased gestation length (GESTLENGTHINCR)<br>• Dystocia (DYSTOCIA)
Parturition, lactation and weaning in dF1 and dF2<br>• Decreased live birth index (LIVEBIRTHX)<br>• Decreased number of delivered pups/ litter size (DELIVEREDDECR)<br>• Post-natal loss (POSTNATALLOSS)<br>• No milk in stomach of pups (NOMILK)<br>• Decreased body temperature of pups (HYPOTHERM)<br>• Hypoactivity in pups (HYPOACT)<br>• Decreased anogenital index in males (AGDMALEDECR)<br>• Increased anogenital index in females (AGDFEMINCR)<br>• Nipple/ areolae retention (NIPPLERET)	***Sexual maturation in dF1*** [*]<br>• Post-weaning loss (POSTWEANLOSS)<br>• Delayed balano preputial separation (BPSLATE)<br>• Precocious balano preputial separation (BPSEARLY)<br>• Delayed vaginal opening (VOLATE)<br>• Precocious vaginal opening (VOEARLY)<br>• Delayed first estrus (1STESTRLATE)<br>• Precocious first estrus (1STESTREARLY)<br><br>[*] These parameters are typically not investigated in the dF2 because OECD TG 443 does not require their measurement (dF2 is usually kept until weaning)

Table S3. Jaccard (***J***) coefficients for all effect-effect pairs with p ≤ 0.05 (Fisher's Exact test) within the same and across different generations and life stages are sorted by descending ***J*** coefficients. Stronger associations, indicated by higher ***J*** coefficients, are displayed at the top of the table, while weaker ones, with lower ***J*** coefficients, are shown at the bottom. Statistical significance for J coefficients is denoted by * (0.1 < p ≤ 0.05), ** (0.001 < p ≤ 0.01) and, *** (p ≤ 0.001).

**Effect**	**Associated effect**	***J* coefficient**	**Logical coherence**	**Explanation for logical coherence**
aF0.EPIDIHISTO	aF1.EPIDIHISTO	0.857***	Yes	Same effect in different generations at same life stage
dF1.POSTNATALLOSS	dF2.POSTNATALLOSS	0.769***	Yes	Same effect in different generations at same life stage
P1.PREIMPLOSS	dF2.DELIVEREDDECR	0.750***	Yes	↓ number of implants results in ↓ number of deliveries
dF1.NOMILK	dF1.HYPOTHERM	0.667***	Yes	↓ body temperature in suckling results from ↓ milk intake
P0.PREIMPLOSS	dF1.DELIVEREDDECR	0.636***	Yes	↓ number of implants results in ↓ number of deliveries
aF0.TESTISHISTO	aF0.EPIDIHISTO	0.625***	Yes	Anatomically linked, functionally linked (e.g., sperm production), shared endocrine pathways
aF0.TESTISHISTO	aF1.TESTISHISTO	0.625***	Yes	Same effect in different generations at same life stage
aF1.TESTISHISTO	aF1.EPIDIHISTO	0.625***	Yes	Anatomically linked, Functionally linked (sperm production), shared endocrine pathways
aF0.EPIDIHISTO	dF2.AGDFEMINCR	0.600**	Yes	Shared endocrine pathways
aF1.TESTISHISTO	dF2.AGDFEMINCR	0.600**	Yes	Shared endocrine pathways
aF1.EPIDIHISTO	dF2.AGDFEMINCR	0.600**	Yes	Shared endocrine pathways
aF0.EPIDIHISTO	aF1.TESTISHISTO	0.556***	Yes	Anatomically linked, functionally linked (e.g. sperm production), shared endocrine pathways
aF1.FOLLICLEDECR	P1.FERTXDECR	0.545***	Yes	Anatomically linked, functionally linked, ↓ number of ovarian follicles results in ↓ female fertility
dF1.DELIVEREDDECR	dF2.DELIVEREDDECR	0.526***	Yes	Same effect in different generations at same life stage
aF0.TESTISHISTO	aF1.EPIDIHISTO	0.500***	Yes	Same effect in different generations at same life stage
dF1.BPSLATE	dF1.VOLATE	0.474***	Yes	Same effect in different sexes of same generation: delayed onset of puberty
P0.POSTIMPLOSS	P1.POSTIMPLOSS	0.455**	Yes	Same effect in different generations
aF0.SPERMMOTIL	dF2.DELIVEREDDECR	0.438**	Yes	↓ sperm motility results in ↓ number of offspring

aF0.SPERMMOTIL	aF1.SPERMMOTIL	0.421***	Yes	Same effect in different generations
dF1.DELIVEREDDECR	P1.PREIMPLOSS	0.421*	Yes	↓ number of implants results in ↓ number of deliveries
aF0.SPERMMORPH	aF1.SPERMMORPH	0.417***	Yes	Same effect in different generations
aF0.SPERMATIDCOUNT	aF0.SPERMPRODRATE	0.400**	Yes	↓ sperm production results in ↓ number of spermatids
P0.DYSTOCIA	P1.DYSTOCIA	0.400*	Yes	Same effect in different generations
aF0.EPIDIWGT	aF1.SPERMMOTIL	0.400***	Yes	Epididymis pathology results in ↓ sperm motility
aF0.EPIDIWGT	aF1.EPIDIWGT	0.400***	Yes	Same effects in different generations
P1.GESTLENGTHINCR	dF2.AGDFEMINCR	0.400*	Yes	Shared endocrine pathways
aF0.TESTISHISTO	dF2.AGDFEMINCR	0.400*	Yes	Shared endocrine pathways
aF0.MAMMARYHISTOF	dF2.LIVEBIRTHX	0.400*	Yes	Shared endocrine pathways
P1.GESTLENGTHINCR	dF2.AGDFEMINCR	0.400*	Yes	Shared endocrine pathways
aF0.MAMMARYHISTOF	P1.GESTLENGTHINCR	0.400*	Yes	Shared endocrine pathways
dF1.BPSLATE	P1.PREIMPLOSS	0.400*	Yes	Shared endocrine pathways
aF1.TESTISWGT	aF1.EPIDIWGT	0.385***	Yes	Shared endocrine pathways
aF0.TESTISHISTO	aF0.EPIDIWGT	0.375***	Yes	Anatomically linked, functionally linked (e.g., sperm production), shared endocrine pathways
P1.POSTIMPLOSS	dF2.POSTNATALLOSS	0.375*	Yes	Shared endocrine pathways
dF1.POSTNATALLOSS	P1.POSTIMPLOSS	0.357*	Yes	Shared endocrine pathways, both relate to offspring viability
P0.PREIMPLOSS	P1.PREIMPLOSS	0.353*	Yes	Same effects in different generations
aF0.ESTRCYCLIRR	dF1.NOMILK	0.350***	Yes	Shared endocrine pathways
aF0.SPERMATIDRET	aF1.EPIDIWGT	0.333***	Yes	Functionally linked (sperm production), shared endocrine pathways
aF0.EPIDIWGT	aF0.SPERMATIDRET	0.333**	Yes	Functionally linked (sperm production), shared endocrine pathways
aF0.EPIDIWGT	aF0.OVARWGT	0.333**	Yes	Shared endocrine pathways
aF0.EPIDIHISTO	aF1.SPERMMORPH	0.333***	Yes	Functionally linked (sperm production), shared endocrine pathways
aF0.EPIDIWGT	aF0.EPIDIHISTO	0.333**	Yes	Pathology in the same organ of the same generation
P0.POSTIMPLOSS	dF1.DELIVEREDDECR	0.333***	Yes	↓ number of implants results in ↓ number of deliveries
P0.GESTLENGTHINCR	dF2.NOMILK	0.333*	Yes	↑ gestation length might indicate health issues in dam resulting in ↓ nursing behavior

P0.DYSTOCIA	dF2.LIVEBIRTHX	0.333*	Yes	Dystocia results in ↓ offspring viability
P0.GESTLENGTHINCR	P1.GESTLENGTHINCR	0.333*	Yes	Same effect in different generations
P0.DYSTOCIA	P1.GESTLENGTHINCR	0.333*	Yes	Increased gestation length results in dystocia
aF1.ESTRCYCLIRR	P1.GESTLENGTHINCR	0.333*	Yes	Shared endocrine pathways
aF1.TESTISWGT	aF1.TESTISHISTO	0.333***	Yes	Pathology in the same organ of the same generation
aF0.EPIDIHISTO	aF1.SPERMMOTIL	0.333***	Yes	Functionally linked (sperm production), shared endocrine pathways
aF0.SPERMMOTIL	dF2.AGDFEMINCR	0.333*	Yes	Shared endocrine pathways
dF1.AGDFEMINCR	dF2.AGDFEMINCR	0.333*	Yes	Same effect in different generations
aF1.SPERMMOTIL	dF2.AGDFEMINCR	0.333*	Yes	Shared endocrine pathways
P1.POSTIMPLOSS	dF2.DELIVEREDDECR	0.333*	Yes	↓ number of implants results in ↓ number of deliveries
aF0.SPERMMOTIL	P1.PREIMPLOSS	0.312*	Yes	↓ sperm quality results in ↓ implantation rate
aF1.SPERMMOTIL	aF1.SPERMCOUNT	0.308**	Yes	Shared endocrine pathways, both linked to ↓ sperm quality
P0.FERTXDECR	P1.FERTXDECR	0.308*	Yes	Same effect in different generations
aF1.SPERMMORPH	aF1.SPERMMOTIL	0.294***	Yes	Shared endocrine pathways, both linked to ↓ sperm quality
aF0.TESTISHISTO	aF0.SPERMATIDRET	0.286**	Yes	Functionally linked (spermatid production), shared endocrine pathways
aF0.OVARWGT	aF1.OVARWGT	0.286**	Yes	Same effect in different generations
aF0.EPIDIWGT	aF1.SPERMATIDCOUNT	0.286**	Yes	Functionally linked (sperm production), shared endocrine pathways
P0.GESTLENGTHINCR	dF2.POSTNATALLOSS	0.286*	Yes	Increased gestation length results in ↓ offspring viability
aF0.SPERMCOUNT	aF1.EPIDIWGT	0.286**	Yes	Functionally linked (sperm production), shared endocrine pathways
dF1.POSTNATALLOSS	dF1.NOMILK	0.273***	Yes	↓ breast milk intake results in ↓ offspring viability
aF0.EPIDIWGT	aF1.TESTISWGT	0.273**	Yes	Anatomically linked, Functionally linked (sperm production), shared endocrine pathways
aF0.PROSTSVWGT	aF1.PROSTSVWGT	0.273**	Yes	Same effect in different generations
P0.GESTLENGTHINCR	dF1.NOMILK	0.273**	Yes	Increased gestation length results in health issues of the dam resulting in problems breast feeding
aF0.EPIDIHISTO	dF2.DELIVEREDDECR	0.267*	Yes	Shared endocrine pathways

aF1.TESTISHISTO	dF2.DELIVEREDDECR	0.267*	Yes	Shared endocrine pathways
aF1.EPIDIHISTO	dF2.DELIVEREDDECR	0.267*	Yes	Shared endocrine pathways
aF1.PROSTSVWGT	dF2.DELIVEREDDECR	0.267*	Yes	Shared endocrine pathways
aF0.TESTISHISTO	aF1.SPERMMORPH	0.267**	Yes	Functionally linked (sperm production), shared endocrine pathways
aF1.SPERMMORPH	aF1.EPIDIHISTO	0.267**	Yes	Functionally linked (sperm production), shared endocrine pathways
aF1.FOLLICLEDECR	aF1.ESTRCYCLIRR	0.263**	Yes	Functionally linked (intertwined ovarian and estrus cyclicity)
P0.PREIMPLOSS	P0.POSTIMPLOSS	0.263**	Yes	Shared endocrine pathways, results from death of conceptus
aF0.SPERMMORPH	aF0.SPERMMOTIL	0.258**	Yes	Both effects relate to sperm quality under the control of the same functional units (testis and epididymis) and endocrine pathways
P0.DYSTOCIA	dF1.VOEARLY	0.250**	Yes	Shared endocrine pathways
aF0.EPIDIHISTO	aF0.SPERMATIDRET	0.250**	Yes	Functionally linked (sperm production), shared endocrine pathways
aF0.SPERMMOTIL	aF0.EPIDIHISTO	0.250**	Yes	Functionally linked (sperm production), shared endocrine pathways
dF1.AGDFEMINCR	dF1.1STESTRLATE	0.250*	Yes	Shared endocrine pathways
aF1.SPERMMOTIL	aF1.EPIDIHISTO	0.250**	Yes	Functionally linked (sperm production), shared endocrine pathways
aF0.TESTISHISTO	aF1.SPERMMOTIL	0.250**	Yes	Functionally linked (sperm production), shared endocrine pathways
aF0.TESTISHISTO	aF1.TESTISWGT	0.250**	Yes	Pathology in the same organ of the same generation
aF0.TESTISHISTO	aF1.EPIDIWGT	0.250**	Yes	Anatomically linked, functionally linked (e.g. sperm production), shared endocrine pathways
aF1.SPERMMORPH	aF1.TESTISHISTO	0.250**	Yes	Functionally linked (sperm production), shared endocrine pathways
aF0.EPIDIHISTO	aF1.SPERMCOUNT	0.250**	Yes	Functionally linked (sperm production), shared endocrine pathways
aF1.SPERMCOUNT	aF1.TESTISHISTO	0.250**	Yes	Functionally linked (sperm production), shared endocrine pathways
dF1.DELIVEREDDECR	aF1.PROSTSVWGT	0.240**	Yes	Shared endocrine pathways
dF1.DELIVEREDDECR	aF1.ESTRCYCLIRR	0.240**	Yes	Shared endocrine pathways
aF0.ESTRCYCLIRR	dF1.HYPOTHERM	0.238**	Yes	Irregular estrus cyclicity may indicate disturbed endocrine pathways that diminish nursing behavior

aF1.SPERMMORPH	aF1.SPERMCOUNT	0.235**	Yes	Shared endocrine pathways, both linked to ↓ sperm quality
dF1.DELIVEREDDECR	dF1.POSTNATALLOSS	0.235*	Yes	Both effects relate to decreased offspring viability at parturition and postnatally
dF2.LIVEBIRTHX	dF2.POSTNATALLOSS	0.231*	Yes	Both effects relate to decreased offspring viability at parturition and postnatally
dF2.POSTNATALLOSS	dF2.NOMILK	0.231*	Yes	↓ milk in the stomach of pups results in ↓ offspring viability
P1.GESTLENGTHINCR	dF2.POSTNATALLOSS	0.231*	Yes	Increased gestation length results in ↓ offspring viability
dF1.NOMILK	dF2.POSTNATALLOSS	0.231*	Yes	↓ milk in the stomach of pups results in ↓ offspring viability
aF0.EPIDIHISTO	aF1.EPIDIWGT	0.231*	Yes	Pathology in the same organ in different generations
aF1.SPERMMOTIL	aF1.TESTISHISTO	0.231*	Yes	Functionally linked (sperm production), shared endocrine pathways
aF1.TESTISHISTO	aF1.EPIDIWGT	0.231*	Yes	Anatomically linked, functionally linked (e.g., sperm production), shared endocrine pathways
P0.DYSTOCIA	dF1.NOMILK	0.231*	Yes	Dystocia results in health issues of the dam resulting in ↓ nursing of offspring
dF1.VOEARLY	aF1.ESTRCYCLIRR	0.222**	Yes	Shared endocrine pathways
aF0.OVARHISTO	P1.POSTIMPLOSS	0.222*	Yes	Shared endocrine pathways
aF1.OVARHISTO	P1.POSTIMPLOSS	0.222*	Yes	Shared endocrine pathways
aF0.SPERMATIDRET	aF1.SPERMCOUNT	0.222*	Yes	Shared endocrine pathways, both linked to ↓ sperm quality
aF0.EPIDIWGT	aF1.EPIDIHISTO	0.222*	Yes	Pathology in the same organ
aF0.PROSTSVWGT	aF1.EPIDIHISTO	0.222*	Yes	Anatomically linked, functionally linked (sperm), shared endocrine pathways
dF1.LIVEBIRTHX	dF1.DELIVEREDDECR	0.222**	Yes	Both effects relate to decreased offspring viability at parturition
aF0.ESTRCYCLIRR	dF1.POSTNATALLOSS	0.219*	Yes	Shared endocrine pathways
aF0.SPERMMORPH	aF0.EPIDIHISTO	0.217**	Yes	Functionally linked (sperm production), shared endocrine pathways
aF0.SPERMMORPH	aF1.TESTISHISTO	0.217**	Yes	Functionally linked (sperm production), shared endocrine pathways
aF1.SPERMMORPH	aF1.SPERMATIDCOUNT	0.214**	Yes	Shared endocrine pathways, both linked to ↓ sperm quality

dF1.LIVEBIRTHX	aF1.OVARWGT	0.214*	Yes	Shared endocrine pathways
aF1.SPERMCOUNT	aF1.EPIDIWGT	0.214*	Yes	Functionally linked (sperm production), shared endocrine pathways
aF0.ESTRCYCLIRR	aF1.FOLLICLEDECR	0.214*	Yes	Functionally linked (intertwined ovarian and estrus cyclicity), shared endocrine pathways
aF0.SPERMMOTIL	dF1.DELIVEREDDECR	0.212*	Yes	↓ sperm quality results in ↓ number of offspring
aF0.SPERMMOTIL	aF0.EPIDIWGT	0.211**	Yes	Functionally linked (sperm production), shared endocrine pathways
P0.PREIMPLOSS	aF1.TESTISWGT	0.211*	Yes	Shared endocrine pathways
P0.PREIMPLOSS	aF1.PROSTSVWGT	0.211*	Yes	Shared endocrine pathways
P0.PREIMPLOSS	aF1.ESTRCYCLIRR	0.211*	Yes	↓ implantation results from structural changes in endometrium that depends inter alia on estrogenic and androgenic pathways
aF0.SPERMMORPH	aF1.SPERMCOUNT	0.208**	Yes	Shared endocrine pathways, both linked to ↓ sperm quality
dF1.POSTNATALLOSS	aF1.ESTRCYCLIRR	0.208**	Yes	Shared endocrine pathways
P0.PREIMPLOSS	aF1.FOLLICLEDECR	0.208*	Yes	Shared endocrine pathways, ↓ number of follicles results in ↓ implantation
P0.PREIMPLOSS	dF1.BPSLATE	0.206*	Yes	Shared endocrine pathways
aF0.PROSTSVHISTO	aF0.PROSTSVWGT	0.200*	Yes	Pathology in the same organ of the same generation
aF0.SPERMATIDCOUNT	aF0.CORPLUTDECR	0.200*	Yes	Shared endocrine pathways
aF0.SPERMATIDCOUNT	aF1.SPERMPRODRATE	0.200*	Yes	↓ production rate results in ↓ spermatid count
aF0.EPIDIWGT	aF0.PROSTSVHISTO	0.200*	Yes	Anatomically linked, functionally linked (sperm), shared endocrine pathways
dF1.AGDFEMINCR	aF1.UTERUSWGT	0.200*	Yes	Shared endocrine pathways
aF1.FOLLICLEDECR	aF1.OVARHISTO	0.200**	Yes	Pathology in ovary results in ↓ number of follicles
aF0.TESTISHISTO	dF2.DELIVEREDDECR	0.200*	Yes	Shared endocrine pathways
aF0.SPERMCOUNT	aF0.SPERMATIDRET	0.200*	Yes	↓ sperm count results from ↑ spermatid retention
aF0.SPERMATIDRET	aF1.SPERMMOTIL	0.200*	Yes	Shared endocrine pathways, both linked to ↓ sperm quality
aF1.TESTISWGT	aF1.OVARHISTO	0.200*	Yes	Shared endocrine pathways
aF1.PROSTSVWGT	aF1.MAMMARYHISTOM	0.200*	Yes	Shared endocrine pathways
aF1.PROSTSVWGT	aF1.OVARHISTO	0.200*	Yes	Shared endocrine pathways
aF1.ESTRCYCLIRR	aF1.OVARHISTO	0.200*	Yes	Functionally linked (intertwined ovarian an

				estrus cyclicity), Shared endocrine pathways
aF0.SPERMMOTIL	aF0.TESTISHISTO	0.200**	Yes	Functionally linked (sperm production), shared endocrine pathways
aF0.SPERMMOTIL	aF1.EPIDIHISTO	0.200**	Yes	Functionally linked (sperm production), shared endocrine pathways
aF0.EPIDIWGT	aF1.SPERMMORPH	0.200*	Yes	Functionally linked (sperm production), shared endocrine pathways
aF0.EPIDIWGT	aF1.TESTISHISTO	0.200*	Yes	Functionally linked (sperm production), shared endocrine pathways
aF0.EPIDIHISTO	aF0.PROSTSVWGT	0.200*	Yes	Anatomically linked, functionally linked (sperm), shared endocrine pathways
P0.PERINATMATDEATH	dF1.HYPOTHERM	0.200*	Yes	↑ Perinatal maternal death indicates maternal ill being in surviving dams resulting in ↓ nursing
dF1.LIVEBIRTHX	dF1.HYPOTHERM	0.200*	Yes	Decreased body temperature results in ↓ offspring viability
aF0.SPERMMORPH	aF1.SPERMMOTIL	0.200*	Yes	Shared endocrine pathways, both linked to ↓ sperm quality
aF0.SPERMCOUNT	aF1.SPERMMOTIL	0.200*	Yes	Shared endocrine pathways, both linked to ↓ sperm quality
aF1.SPERMMOTIL	aF1.EPIDIWGT	0.200*	Yes	Anatomically linked, functionally linked (sperm), shared endocrine pathways
aF1.TESTISWGT	aF1.PROSTSVWGT	0.200*	Yes	Shared endocrine pathways, functionally linked
aF1.PROSTSVWGT	aF1.ESTRCYCLIRR	0.200*	Yes	Shared endocrine pathways
P0.FERTXDECR	aF1.FOLLICLEDECR	0.200*	Yes	Anatomically linked, functionally linked, ↓ number of ovarian follicles results in ↓ female fertility
dF1.LIVEBIRTHX	dF1.POSTNATALLOSS	0.192*	Yes	Both effects relate to decreased offspring viability at parturition and postnatally
aF0.SPERMMOTIL	aF1.SPERMMORPH	0.192*	Yes	Shared endocrine pathways, both linked to ↓ sperm quality
aF0.ESTRCYCLIRR	P0.GESTLENGTHINCR	0.190**	Yes	Shared endocrine pathways
aF0.SPERMMOTIL	aF1.TESTISHISTO	0.190*	Yes	Functionally linked (sperm production), shared endocrine pathways
P0.DYSTOCIA	dF1.LIVEBIRTHX	0.188*	Yes	Dystocia decreases offspring viability
dF1.LIVEBIRTHX	dF1.NOMILK	0.188*	Yes	Both effects relate to decreased offspring viability
aF0.SPERMMORPH	dF1.LIVEBIRTHX	0.185*	Yes	Shared endocrine pathways

P0.GESTLENGTHINCR	dF1.POSTNATALLOSS	0.182**	Yes	Increased gestation length results in ↓ offspring viability
aF1.SPERMMOTIL	aF1.SPERMATIDCOUNT	0.182*	Yes	Shared endocrine pathways, both linked to ↓ sperm quality
aF1.SPERMATIDCOUNT	aF1.TESTISWGT	0.182*	Yes	Functionally linked (sperm production), shared endocrine pathways
aF1.SPERMATIDCOUNT	aF1.EPIDIWGT	0.182*	Yes	Functionally linked (sperm production), shared endocrine pathways
aF0.EPIDIWGT	aF1.SPERMCOUNT	0.182*	Yes	Functionally linked (sperm production), shared endocrine pathways
aF0.MAMMARYHISTOF	P0.DYSTOCIA	0.182*	Yes	Shared endocrine pathways
P0.PERINATMATDEATH	P0.DYSTOCIA	0.182*	Yes	Dystocia results in perinatal maternal death
P0.PERINATMATDEATH	dF1.NOMILK	0.182*	Yes	Perinatal maternal death is an indicator of health issues in the dam resulting in problems with lactation
P0.GESTLENGTHINCR	dF1.HYPOTHERM	0.182*	Yes	↑ Perinatal maternal death indicates maternal ill being in surviving dams resulting in ↓ nursing
aF0.SPERMMOTIL	aF1.SPERMCOUNT	0.182*	Yes	Shared endocrine pathways, both linked to ↓ sperm quality
P0.PREIMPLOSS	aF1.EPIDIHISTO	0.176*	Yes	Shared endocrine pathways
aF0.SPERMMORPH	aF0.TESTISHISTO	0.174*	Yes	Functionally linked (sperm production), shared endocrine pathways
aF0.SPERMMORPH	aF1.EPIDIHISTO	0.174*	Yes	Anatomically linked, functionally linked (sperm), shared endocrine pathways
dF1.POSTNATALLOSS	dF1.HYPOTHERM	0.174*	Yes	Both effects relate to decreased offspring viability postnatally
aF0.ESTRCYCLIRR	P0.DYSTOCIA	0.174*	Yes	Shared endocrine pathways
dF1.VOLATE	dF1.1STESTRLATE	0.172***	Yes	Both effects are functionally linked and relate to the same parameter: onset of puberty and sexual maturation
aF0.OVARWGT	dF1.LIVEBIRTHX	0.167*	Yes	Shared endocrine pathways
dF1.DELIVEREDDECR	aF1.EPIDIHISTO	0.167*	Yes	Shared endocrine pathways
aF0.EPIDIHISTO	P0.PREIMPLOSS	0.167*	Yes	Shared endocrine pathways
P0.PREIMPLOSS	aF1.TESTISHISTO	0.167*	Yes	Shared endocrine pathways
P0.DYSTOCIA	dF1.POSTNATALLOSS	0.167*	Yes	Dystocia results in ↓ offspring viability
aF0.ESTRCYCLIRR	aF1.ESTRCYCLIRR	0.167*	Yes	Same effect in different generations at same life stage

dF1.BPSLATE	aF1.EPIDIWGT	0.161*	Yes	Shared endocrine pathways
aF0.EPIDIHISTO	dF1.DELIVEREDDECR	0.160*	Yes	Shared endocrine pathways
dF1.DELIVEREDDECR	aF1.TESTISHISTO	0.160*	Yes	Shared endocrine pathways
dF1.LIVEBIRTHX	aF1.SPERMATIDCOUNT	0.154*	Yes	Shared endocrine pathways
dF1.DELIVEREDDECR	dF1.NOMILK	0.154*	Yes	↓ number of offspring indicative of developmental effects in offspring linked to issues with sucking; ↓ number of offspring indicative of health effects in dams linked to issues in nursing
P0.PREIMPLOSS	dF1.NIPPLERET	0.143*	Yes	Shared endocrine pathways
aF0.SPERMATIDRET	aF1.SPERMMORPH	0.143*	Yes	Shared endocrine pathways, both linked to ↓ sperm quality
aF0.OVARHISTO	P0.FERTXDECR	0.133*	Yes	Pathology in ovaries results in decreased female fertility
aF0.OVARHISTO	aF1.FOLLICLEDECR	0.133*	Yes	Anatomically related: follicular development takes place in ovaries
P0.PREIMPLOSS	aF1.OVARHISTO	0.133*	Yes	Pathology in ovaries results in decreased follicle/ corpora lutea quality resulting in preimplantation loss
dF1.DELIVEREDDECR	dF1.AGDMALEDECR	0.130*	Yes	Shared endocrine pathways
aF0.SPERMMORPH	aF0.EPIDIWGT	0.130*	Yes	Anatomically linked, functionally linked (sperm), shared endocrine pathways
P0.FERTXDECR	aF1.OVARHISTO	0.125*	Yes	Pathology in ovaries results in ↓ female fertility
dF1.POSTNATALLOSS	aF1.MAMMARYHISTOF	0.100*	Yes	Mammary pathology results in functional problems with breast feeding
aF0.SPERMMORPH	dF1.BPSEARLY	0.095*	Yes	Shared endocrine pathways
dF1.DELIVEREDDECR	dF1.NIPPLERET	0.091*	Yes	Shared endocrine pathways

Table S4. Statistical parameters for the distribution of Jaccard coefficients (***J***) in the categories. SD, standard deviation. Min, minimum. Q1, 25th percentile. Q3, 75th percentile. Max, maximum.

Figure	Category	Mean	SD	Min	Q_1	Median	Q_3	Max	N blue	N overlap	N orange	*N total*
**3**	All	0.278	0.134	0.091	0.200	0.231	0.333	0.857	777	1267	960	3004
	Same generations	0.252	0.107	0.091	0.200	0.226	0.263	0.667	239	513	240	992
	Different generations	0.290	0.144	0.095	0.200	0.239	0.333	0.857	538	754	720	2012
	Same effects	0.433	0.183	0.167	0.327	0.400	0.472	0.857	83	65	52	200
	Different effects	0.264	0.120	0.091	0.200	0.222	0.309	0.750	694	1202	908	2804
**4**	dF1 × dF2	0.465	0.237	0.231	0.308	0.430	0.587	0.769	25	6	20	51
	aF0 × dF2	0.377	0.129	0.200	0.300	0.400	0.419	0.600	24	10	36	70
	dF1 × P1	0.393	0.033	0.357	0.379	0.400	0.411	0.421	21	18	14	53
	P1 × dF2	0.418	0.185	0.231	0.333	0.375	0.400	0.750	58	16	62	136
	P0 × P1	0.364	0.054	0.308	0.333	0.343	0.388	0.455	21	20	17	58
	P0 × dF2	0.317	0.027	0.286	0.310	0.333	0.333	0.333	8	7	11	26
	aF1 × P1	0.367	0.164	0.222	0.278	0.333	0.439	0.545	10	6	10	26
	aF0 × P1	0.312	0.089	0.222	0.267	0.313	0.356	0.400	9	5	16	30
	aF1 × dF2	0.389	0.166	0.267	0.267	0.300	0.533	0.600	20	4	37	61
	aF0 × aF1	0.282	0.140	0.133	0.200	0.231	0.310	0.857	160	241	214	615
	P0 × aF1	0.182	0.034	0.125	0.167	0.200	0.211	0.211	32	96	44	172
	P0 × dF1	0.244	0.128	0.143	0.182	0.200	0.250	0.636	56	56	108	220
	dF1 × aF1	0.188	0.043	0.100	0.161	0.200	0.218	0.240	40	144	31	215
	aF0 × dF1	0.203	0.074	0.095	0.165	0.199	0.224	0.350	39	88	64	191
	aF0 × P0	0.169	0.022	0.133	0.300	0.174	0.182	0.190	15	37	36	88
**5**	All	0.278	0.134	0.091	0.200	0.231	0.333	0.857	777	1267	960	3004
	$J > 0.30$	0.386	0.141	0.200	0.308	0.333	0.400	0.769	196	92	223	511
	$J < 0.24$	0.246	0.114	0.091	0.186	0.211	0.261	0.857	342	662	497	1501
**6**	All	0.278	0.134	0.091	0.200	0.231	0.333	0.857	777	1267	960	3004
	P0 × dF1	0.244	0.128	0.143	0.182	0.200	0.250	0.636	56	56	108	220
	P1 × dF2	0.418	0.185	0.231	0.333	0.375	0.400	0.750	58	16	62	136
	aF0 × P0	0.169	0.022	0.133	0.167	0.174	0.182	0.190	15	37	36	88
	aF1 × P1	0.367	0.164	0.222	0.278	0.333	0.439	0.545	10	6	10	26
**7**	aF0 × aF1	0.282	0.140	0.133	0.200	0.231	0.310	0.857	160	241	214	615
	P0 × P1	0.364	0.054	0.308	0.333	0.343	0.388	0.455	21	20	17	58
	dF1 × dF2	0.465	0.237	0.231	0.308	0.430	0.587	0.769	25	6	20	51
